# Similarity is associated with where repeated-event memories fall on the semantic–episodic continuum

**DOI:** 10.3758/s13421-025-01729-6

**Published:** 2025-05-20

**Authors:** Oliver R. Bontkes, Daniela J. Palombo, Eva Rubínová

**Affiliations:** 1 Department of Psychology, The University of British Columbia, Douglas T. Kenny Building, 2136 West Mall, Vancouver, BC V6 T 1Z4 Canada; 2 The School of Psychology, University of Aberdeen, William Guild Building, Old Aberdeen, AB24 3 FX UK

**Keywords:** Autobiographical memory, Episodic memory, Repeated events, Semantic memory, Semantic–episodic continuum, Similarity

## Abstract

Memories of repeated events are one form of memory thought to be intermediate on a proposed semantic–episodic continuum. However, it is not yet understood where repeated-event memories fall on this continuum, and which factors may be associated with greater or lesser reliance on episodic and semantic memory during recall. We investigated similarity amongst instances of repeated events as one factor which may be associated with where repeated events fall on the semantic–episodic continuum. In two preregistered studies, we asked participants to recall three repeated-event memories from their own lives (*N*_1_ = 97 participants, 291 memories; *N*_2_ = 419 participants, 1,257 memories) and report on the similarity amongst instances as well as the degree to which they relied on semantic memory, a single episode, and a mix of episodes in their recall of each event. In line with our predictions, similarity was positively correlated with reliance on semantic memory in both studies. In Study 2, similarity was negatively correlated with reliance on a single episode. We also conducted exploratory latent profile analyses using our three memory reliance variables, revealing three types of repeated-event memories. In both studies, similarity of place and emotional arousal were each associated with different memory profiles. Our findings highlight the importance of considering similarity in basic and applied repeated-event memory research, as different conditions of similarity (e.g., low vs. high) can manifest in different patterns of reliance on episodic and semantic memory.

**Supplementary Information:**

The online version contains supplementary material available at 10.3758/s13421-025-01729-6.

In 1972, Tulving proposed a distinction between episodic memory, which refers to memory of a single event that is localized in place and time, and semantic memory, which involves general factual knowledge that is detached from the original spatiotemporal context of acquisition. Although hints of this distinction have been formulated in various ways since the time of Aristotle (see Herrmann, 1982), Tulving’s delineation has dominated research on declarative memory since its conception (Renoult et al., 2019). In the ensuing 50 years, a wealth of research has demonstrated evidence to support a distinction between episodic and semantic memory (e.g., Levine et al., 2002; Vargha-Khadem et al., 1997; see also Renoult & Rugg, 2020; Tulving, 1983), though critiques of this distinction have also emerged (e.g., Addis & Szpunar, 2024; De Brigard et al., 2022; McKoon et al., 1986). Some alternative theoretical approaches at that time favored the idea that episodic and semantic memory emerge from a single system, and that dissociations between memory types are better explained by differences in the types of processes engaged as opposed to different memory systems (i.e., processes vs. systems views of memory; see Roediger et al., 1999, for review).

It is sometimes overlooked in the literature that Tulving noted some interdependence between semantic and episodic memory; for example, semantic knowledge is often a generalization of information from multiple episodes, and episodic memories can be organized by semantic associations (Tulving, 1983; also see Addis & Szpunar, 2024; De Brigard et al., 2022; Irish & Piguet, 2013; Renoult & Rugg, 2020). Further research highlighted that many memories do not fall neatly into either category (Greenberg & Verfaellie, 2010; Renoult et al., 2012) and that a more apt conceptualization may be a continuum with semantic and episodic memory at the two extremes (also see Addis & Szpunar, 2024; De Brigard et al., 2022; Renoult & Rugg, 2020; Rubin, 2022, for other viewpoints).^1^

This proposed semantic–episodic continuum generated a new interest in “personal semantics,” a concept that has been used to encapsulate intermediate forms of memory, including autobiographical facts, autobiographically significant concepts, self-knowledge, and repeated events (Renoult et al., 2012). These forms of memory are largely detached from their context of acquisition, like semantic memory, but they are personal, like episodic memory (Renoult et al., 2012). For example, one’s memory of their brother’s favorite soccer player is unlikely to be accompanied by episodic memory for the time and place in which this knowledge was acquired, but it is nonetheless personally relevant to the rememberer. Supportive of the continuum idea, neuroimaging work has shown that memories of general facts (facts about the world), autobiographical facts (facts about the self), repeated events (sequences of similar situations), and episodic memories (unique events) activate a common neural network, but that activity in this network increases when moving from general facts to autobiographical facts, from autobiographical facts to repeated events, and from repeated events to unique events (Tanguay et al., 2023; also see Levine et al., 2004; Maguire & Mummery, 1999; Renoult et al., 2019; Svoboda et al., 2006).^2^

In the present study we focus on one form of personal semantic memory: repeated-event memory (Renoult et al., 2012). Repeated-event memories, such as those pertaining to soccer games, vacations, or lectures, are common and significant in human experience (see Barsalou, 1988; Neisser, 1981). Empirical research has frequently associated repeated-event memory with semantic memory (e.g., Conway & Pleydell-Pearce, 2000) and less frequently with episodic memory.

Developmental accounts have highlighted the importance of scripts in repeated-event recall, which are a form of semantic memory (Farrar & Boyer-Pennington, 1999; Farrar & Goodman, 1992). Scripts include generalized, abstracted knowledge about the people, actions, objects, and the usual temporal unfolding of events—for example, how soccer practice might usually proceed from warm-up, to drills, then to a game (see Fivush, 1984; Schank & Abelson, 1977). Indeed, one well-established finding is that children are more likely to report details that are consistent across instances of repeated events, and less likely to report details that are variable across instances or that are unique to a single episode, supporting the idea that individuals abstract generalized scripts from repeated events (Brubacher et al., 2011; Connolly & Lindsay, 2001; Farrar & Goodman, 1992; Fivush, 1984; Hudson & Nelson, 1986; Sims & Morton, 2021; Woiwod et al., 2019). Still, details about instances that deviate significantly from a repeated-event script can be recalled accurately, especially when a retrieval cue specific to the instance is provided (Brubacher et al., 2011; Connolly et al., 2016; Farrar & Boyer-Pennington, 1999; Farrar & Goodman, 1992). Similar findings have been reported in adults (Deck & Paterson, 2020; Dilevski et al., 2020; Maclean et al., 2018; Theunissen et al., 2017; Willén et al., 2014; see Dilevski et al., 2021, for review), including in studies using memories from participants’ own lives (e.g., vacations and dental visits; Ece & Gülgöz, 2021; Willén et al., 2014). This effect may be due to a selective attention to script-discrepant information (Farrar & Goodman, 1992) either during encoding, retrieval, or both.

Repeated-event memory also has parallels with episodic memory. From a continuum perspective, repeated-event memories appear similar to episodic memories in their contextual—particularly spatial—specificity, though details about the timing of repeated-event memories are inherently less specific than for episodic memories. Because repeated-event memories are often accompanied by memory of place, such as the location where soccer practice always occurs, their recollection often involves a field/first-person or observer/third-person perspective (Rubin & Umanath, 2015). These features contribute to a sense of mental time travel or reliving (Renoult et al., 2012; Rubin & Umanath, 2015)—hallmarks of episodic memory. Indeed, Tanguay et al. (2023) recently showed that repeated events and single/unique episodic memories did not differ in their reliance on scenes, although repeated events did score lower on other phenomenological features, such as visual detail and self-relevance (also see Addis et al., 2004; Holland et al., 2011). Both single/unique and repeated-event memories have shared neural correlates, particularly in the hippocampus (Addis et al., 2004; Eichenbaum, 2004; Holland et al., 2011; Levine et al., 2004; St-Laurent et al., 2009; Tanguay et al., 2023), with repeated events sharing more with single/unique events at the neural level than with general or personal facts (Renoult et al., 2012; Tanguay et al., 2023).^3^ Based on these phenomenological parallels and neural correlates, some propose that episodic memory ought to be reconceptualized as “event memory,” which is rooted in scene construction and includes both repeated and single event memories, amongst other forms of recall not traditionally considered episodic (Rubin & Umanath, 2015; also see Rubin, 2022). Spatial location is of central importance to this proposed reconceptualization as it facilitates the binding of event features in memory, which is argued to be a necessary condition for a sense of reliving (Rubin & Umanath, 2015).

The foregoing research highlights both semantic and episodic contributions to repeated-event memory. These findings raise an intriguing research question: Are there factors that influence where repeated-event memories fall on a semantic–episodic continuum? Arguably, the position along this continuum is not static; instead, repeated-event memory might shift along this continuum depending on characteristics of the recalled event. Here, we focus on similarity, and we argue that episodic and semantic memory are differentially engaged depending on the degree of similarity between repeated-event instances.

To our knowledge, there has been no research into whether repeated-event memories vary in their use of semantic and episodic memory during recall and, critically, whether this use might vary according to the similarity amongst instances of repeated events. Nonetheless, prior research provides a basis for the notion that similarity is important in shaping repeated-event recall. One laboratory study compared high versus low similarity repeated events to gauge how the overall similarity amongst all repeated-event instances affected recall in children (Danby et al., 2019). Findings from this study indicated that children who experienced the highly similar repeated event had stronger repeated-event scripts, while children who experienced the low-similarity repeated event were more accurate in their episodic reports (i.e., they were more accurate when attributing details to the instance in which they occurred; Danby et al., 2019; also see Johnson et al., 1993). However, the study design was constrained in its ability to directly compare the impact of similarity on memory across conditions as the conditions differed in ways other than similarity, including difficulty regarding the amount of novel information to remember. Given these limitations, the effect of similarity on memory for repeated events ought to be investigated further.

We seek to fill this gap in the literature by investigating whether similarity is associated with the relative contributions of semantic and episodic memory to repeated-event memory. Our research question is of theoretical and practical significance. At a theoretical level, this work could deepen our understanding of the factors that influence how individuals integrate or differentiate their experiences based on the degree of overlap with other experiences. This, in turn, could inform models of memory. Indeed, since much of the empirical literature on repeated-event memory applies a script (i.e., semantic memory) perspective, it is possible that the manipulations used affected participants’ memories in ways that the script-focused theoretical framework could not capture, such as how contributions from episodic memory systems may affect the phenomenological experience of different repeated-event memories (see, e.g., Neisser, 1981; Rubin & Umanath, 2015). At a practical level, repeated-event memory research has historically been focused on understanding the capacity of individuals to report accurately on single instances of repeated events, with the goal of understanding the reliability of witness testimony in criminal justice settings where such reporting is common (see Woiwod et al., 2019). Investigating how similarity might be associated with the relative use of semantic and episodic memory when recalling repeated events could ultimately yield valuable insight about the ability of individuals to report reliably on different aspects of their memories in these applied contexts.

The theoretical and practical significance of our study is enhanced by our decision to employ a naturalistic observational design, departing from the thrust of prior studies on repeated-event memory, which have used primarily experimental designs. Although the designs adopted in prior research allow for greater experimental control and permit analyses of variables such as accuracy—a major interest of the literature due to its applied focus—they potentially lack generalizability to repeated-event memories formed under more naturalistic and variable circumstances (see Bauer, 2023). An investigation into the natural diversity of repeated-event memories could shed light on the ways that repeated events vary under more real-world conditions. This, in turn, could inform future experimental work on repeated-event memory—for example, by inspiring manipulations that more closely mimic the natural variability of repeated events. Adopting a naturalistic design could further facilitate this inquiry because most repeated-events studies instill new repeated-event memories in participants, which could potentially be recalled differently from the more remote memories that tend to be probed in legal cases.

In the present study, we asked adult participants to generate examples of repeated events from their own lives and report on the similarity among instances of each repeated event. We then asked participants to report the degree to which they relied on episodic memory and semantic memory during recollection of each repeated event. We operationalized episodic memory as a specific event that the participant experienced and that occurred just once over the course of a few hours; in other words, a single instance of a repeated event. We operationalized semantic memory as general facts that could be applied to more than one episode or that may not apply to any episode in particular. We predicted that repeated-event similarity would be positively correlated with reliance on semantic memory (H1; semantic hypothesis) and negatively correlated with reliance on episodic memory (H2; episodic hypothesis). Given the dearth of research on repeated-event memory using naturalistic designs, a secondary aim of our study was to capture the natural diversity of repeated-event memories. To do so, we conducted additional exploratory analyses focused on identifying whether there are different types of repeated-event memories based on distinct patterns of reliance on semantic memory, a single episode, and a mix of episodes. Finally, we conducted further explorations of the variables associated with the different types of repeated events that emerged from profile analyses. We report findings from a preregistered Study 1 (Bontkes et al., 2023a; https://osf.io/3avyx) and preregistered replication Study 2 (Bontkes et al., 2023b; https://osf.io/d9ph5).

## Method

Data cleaning and analysis were done using R (Version 4.3.1; R Core Team, 2023). We report how we determined our sample size, all data exclusions, all manipulations, and all measures in the study. Preregistrations, materials, data, and scripts are available on the Open Science Framework (OSF; Study 1: https://osf.io/3avyx; Study 2: https://osf.io/d9ph5; data and materials: https://osf.io/ad65y and https://osf.io/hxrc6). We also performed a variety of supplementary analyses, and the method and results for these analyses are available in our Supplementary Material (https://osf.io/hxrc6).

### Participants

#### Study 1

Study 1 was approved by the University of British Columbia (UBC) Research Ethics Board. We compensated participants with one course credit for their participation.

##### Inclusion criteria

To be eligible to participate in the study, participants had to be fluent in English and be between 18 and 35 years of age. Our decision to focus on younger participants instead of older participants was primarily practical, as we were recruiting from university human subject pools, which are predominantly composed of younger people.

##### Exclusion criteria

Per our preregistration, we excluded participants if their responses were incomplete, if they failed our attention check, or if they failed two out of our three comprehension checks. We also added an additional (not preregistered) exclusion criterion where we eliminated duplicate participants.^4^ Specifically, we retained participants’ first attempt at the survey only and eliminated all subsequent attempts, regardless of whether the first attempt was complete or not, as we were unsure whether repeated survey attempts would affect the quality of participants’ responses.

##### Sampling plan

We preregistered our first study after collecting 127 responses to our survey but before any observation of the data (i.e., data were not yet cleaned or examined by the researchers). To determine the appropriate sample size for Study 1, we preregistered a power simulation for our measures of semantic (H1) and episodic reliance (H2) based on a pilot sample of 20 randomly selected participants from the initial sample, after applying exclusions. Depending on the result of the power simulation, we planned to either proceed to full analysis with the remaining data we had already collected or recruit more participants to meet the target sample. Considering uncertainty regarding exclusions, we planned to recruit over the target sample to ensure that our target would be met in the next wave of data collection, and we planned to retain data from any participants over the target sample.

##### Pilot sample and power analysis

From our initial sample of 127 participants, we eliminated 37 duplicate identifiers, seven incomplete responses, two participants who failed our attention check, eight participants who responded incorrectly to two or more of our three comprehension checks, and two participants who were outside of the required age range (18 to 35; further details of the data cleaning procedures are provided in the Supplementary Material, available on OSF: https://osf.io/ad65y/). We arrived at an initial sample of 71 participants, out of which we randomly selected 20 participants as our pilot sample for power analysis (14 cisgender women and six cisgender men), with an average age of 20.50 years (*SD* = 2.84).

To simulate data for power analysis related to H1, we computed a correlation matrix, mean values, and standard deviations for ratings of similarity and reliance on semantic memory for each of the three repeated events reported by each participant in our pilot sample. Using the largest standard deviation and the correlation matrix, we computed the covariance matrix. We set our initial target sample to *N* = 100 and then generated 5,000 datasets using the “rmvnorm()” function from the *SimDesign* package (Chalmers & Adkins, 2020). To arrive at a power estimate, we computed the repeated-measures correlation for each dataset and computed the proportion of significant results (i.e., *p* < 0.05). We kept changing the value of *N* in the simulated samples until we arrived at 90% power. The estimate of our target sample for the semantic hypothesis was 102 participants. We then repeated the procedure with ratings of reliance on episodic memory (H2). The estimate of our target sample for the episodic hypothesis was 69 participants.

##### Sample

In order to reach our target sample of 102 participants while accounting for potential exclusions, we recruited 113 additional participants from the UBC human participants pool. After data cleaning: eliminating 43 duplicate identifiers, 12 incomplete responses, four participants who failed attention checks, and eight participants who failed comprehension checks in addition to the exclusions reported for the pilot sample above, our total sample was 97 participants. Note that data from the randomly selected pilot participants were excluded from the final sample after applying other exclusions, to ensure there were no duplicates in the pilot sample and the Study 1 sample. Participants in the final sample had a mean age of 20.53 years (*SD* = 2.22), and there were 75 women (70 cisgender, four preferred to self-describe and one preferred not to answer whether they were cisgender or transgender), 21 men (20 cisgender and one transgender), and one participant who chose not to answer our gender questions. On average, participants had completed 14.13 years of education (*SD* = 1.32). Twenty-two participants indicated being currently diagnosed with a mental health condition (e.g., depression or anxiety), with 70 participants indicating having no diagnosis and five participants preferring not to answer. We did not collect demographic data on race or ethnicity. The power estimate of the final sample was slightly under our preregistered target power (~ 88%), but we decided to proceed with analyses as the recruitment window had closed and we gauged that there was only a trivial difference between 88% power and 90% power.

#### Study 2

Study 2 was a preregistered replication approved by the UBC Research Ethics Board and the University of Aberdeen Research Ethics Board.^5^ We compensated participants with one course credit for their participation. The purpose of Study 2 was to perform a direct replication of Study 1 to ensure the robustness and reliability of our findings. Furthermore, because we had somewhat ambiguous results in Study 1 with regards to H2—a nonsignificant effect which trended in the hypothesized direction (see Self-Report Analyses in the Results section)—we wanted to determine if the null result was a real null effect or just an issue with power. Finally, in Study 1 we observed larger effect sizes for similarity of place than overall similarity (see Fig. S2), so we wanted to further explore the relevance of similarity of place in a larger sample to examine whether this pattern of results replicated.

##### Inclusion and exclusion criteria

We used the same criteria as in Study 1 and added a step where we excluded identifiers that were already in the Study 1 sample (not preregistered).

##### Sampling plan and power analysis

We preregistered a replication study after analyzing the results from Study 1 and before collecting any data. We ran a simulated power analysis using data related to H2 (i.e., similarity and reliance on episodic memory), which yielded the smaller effect size (see Results; Bontkes et al., 2023b). This power simulation indicated that a sample size of 345 participants would be needed to reach 90% power. We again planned to oversample due to uncertainty regarding exclusions, and we planned to retain any data beyond our target sample. Note that we began participant recruitment for Study 2 after submitting our replication preregistration.

##### Sample

Recruitment through UBC led to 493 responses. After eliminating 136 duplicate identifiers, data from a further 81 participants were eliminated: 23 incomplete responses, 19 participants failed attention checks, 32 participants failed comprehension checks, and one participant had already participated in Study 1; after a manual data check, we eliminated data from one participant who provided invalid responses (random text instead of repeated-event memories) and five additional duplicate responses.

Recruitment through Aberdeen led to 183 responses. We eliminated one duplicate identifier, nine incomplete responses, data from 15 participants who failed attention checks, and 15 who failed comprehension checks. Combining the two recruitment sites, there were 419 participants in our final sample with a mean age of 20.35 years (*SD* = 2.02), composed of 342 women (339 cisgender, one preferred to self-describe, and two preferred not to answer whether they were cisgender or transgender), 69 men (68 cisgender and one preferred to self-describe), seven nonbinary participants, and one person who chose not to answer our gender questions. On average, participants had completed 13.87 years of education (*SD* = 1.74). Seventy-six participants indicated being currently diagnosed with a mental health condition (e.g., depression or anxiety), with 327 participants stating they have no such diagnosis and 16 participants preferring not to answer this question. In line with Study 1, we did not collect demographic data on race or ethnicity.

### Procedure

Participants were told that the purpose of the study was to learn more about how people recall repeated events. We specified that participants would be asked to generate, recall, and describe repeated events from their own lives and complete a series of questionnaires that pertain to their health history, mood, and personality. Participants who provided consent were screened for English language ability (see inclusion criteria), responded to a series of demographic questions (age, gender, and mental health history; all questions were optional), and completed the first attention check.

In the first phase, participants were told to generate three examples of repeated events from their own lives. We specified that an *event* was a specific activity that lasts no more than a few hours. We specified that *repeated* meant that this activity occurs at least once every 2 weeks and that it has occurred more than five times (e.g., going to the gym, attending lectures for PSYC 100 course, playing a soccer game on the weekend). After providing a list of examples, participants then answered whether they experience these sorts of events in their own lives and completed the first comprehension check asking them to select which kind of event they were asked to generate, with a correction prompt provided if they were not correct. Participants then listed three distinct repeated events that they experienced in their own lives.

In the next phase, participants were shown their first named repeated event and asked to spend 15 s retrieving their memory of the repeated event in as much detail as possible. Participants subsequently rated the phenomenology of their memory (i.e., vividness, visual detail, other sensory detail, emotionality, arousal, and personal relevance of the event) on a sliding scale from 0 to 10, except for emotionality that was rated on a sliding scale from − 5 to 5 (questions were adapted from D’Argembeau & Van der Linden, 2008; Wardell et al., 2021). Participants were then asked to write a narrative of their repeated-event memory in as much detail as possible with a minimum requirement of 150 characters. Participants cycled through all three repeated events (in a fixed order) before moving on to the next set of questions.

In the final phase, we asked participants to answer some questions on the timing and similarity for each repeated event they imagined. Timing questions included the frequency of occurrence of the repeated event in the past month, the average number of days between each instance of the repeated event, the first day they experienced the repeated event, and the last day they experienced the repeated event. For the first and last day, participants were allowed to indicate uncertainty in the exact day, month, or year. Participants rated overall similarity, similarity of place, and similarity of people across instances of the repeated event using a sliding scale from 0 to 10. Our decision to explore similarity of place is supported by Rubin and Umanath’s (2015) work, which emphasizes the importance of spatial location in the phenomenology associated with episodic/event memory. Our choice to include similarity of people was further motivated by an effort to explore how salient stimuli which might vary between instances of repeated events might be associated with how repeated events are remembered. Indeed, repeated-event scripts are thought to contain information on people present during an event (Schank & Abelson, 1977). Participants could skip questions about the similarity of people by indicating that they experienced the repeated event alone.

The next set of questions probed how participants constructed their memory of the repeated event, and specifically whether they pictured episodes or used their knowledge or expectations of the event. An episode was defined as ​​a single memory of a specific event that occurred just once over the course of a few hours (and not longer than a day); that is, the memory of a single instance of a repeated event. Knowledge or expectations were defined as general facts that could be applied to more than one episode or that may not apply to any episode in particular. To illustrate the contrast, we provided participants with an example of each type of memory, which were as follows: “When I went to the gym last Tuesday, I ran on the treadmill for 3 km” for a single episode, versus “Usually, when I go to the gym I lift weights” or “I’ve learned that doing less reps with more weight is good for building muscle” for knowledge or expectations. We then asked participants to answer two comprehension checks related to our operationalizations of an episode and knowledge or expectations. If participants answered incorrectly, they were reminded of the correct definition.

Once completing these comprehension checks, we asked participants to rate the degree that they believed they were relying on one episode in their memory of the repeated event using a sliding scale from 0 to 10. Participants also received a reminder of what an episode is in the description of the question. If participants indicated that they relied on a single episode to some degree, we asked follow up questions on what made this episode significant (emotionality, it was the first experience, it was the most recent experience, it was distinct/different from how the event usually goes, or “Other” with text box for a description of the reason). We instructed participants to select “Other” and type NA in the text box if they did not believe the episode they recalled was significant in any way. If participants indicated that the episode was distinct/different from how the event usually goes, we asked them to describe what made that particular episode distinct from how the event usually goes.

We then asked participants to rate the degree to which they believed they were relying on their knowledge or expectations and the degree to which they believed they were mixing memories of several episodes in their recall (i.e., whether they believed they were picturing multiple episodes of this event, and if so to what degree). Both of these questions were rated on a scale from 0 to 10. We also asked participants if they have any other comments they wished to share regarding how they were constructing their memory of the repeated event.

Participants then answered the timing, similarity, and event construction questions for their second and third repeated-event memories. They then completed a series of questionnaires, ancillary to the present study goals, including the Vividness of Visual Imagery (VVIQ; Marks, 1973), the Center for Epidemiological Studies-Depression scale (CES-D; Radloff, 1977), and the shortened version of the Spielberger (Spielberger et al., 1983) State-Trait Anxiety Inventory (STAI; Zsido et al., 2020). At the end of the survey, we debriefed the participants about the study.

Verbatim instructions and questions presented to participants in the survey are included in the Supplementary Material. The median number of minutes it took participants to complete the survey was 44 min for Study 1 and 37 min for Study 2.

### Data analysis

#### Self-report hypotheses

Our primary method of investigating whether similarity is associated with where repeated events fall on the semantic–episodic continuum was to analyze the correlations between similarity and reliance on semantic memory and a single episode in memory, though we also explored the relationship between other similarity variables and reliance variables in our Supplementary Material (see Fig. S2). Individual repeated-event memories were the unit of observation in all our analyses, meaning there were three observations per participant. For our preregistered hypotheses related to similarity and self-reported measures of reliance on knowledge or expectations (i.e., semantic reliance) and single episode reliance, we used the *rmcorr* package to conduct a repeated measures correlation using bootstrapping, which ensured that we controlled for the dependencies in our data due to repeated observation and that our analyses were robust to any potential violations of normality and/or homogeneity of variance (Bakdash & Marusich, 2017). Note that degrees of freedom in repeated measures correlation is calculated as *N*(*k* − 1) − 1, where *N* is the sample size and *k* is the average number of observations per participant (Bakdash & Marusich, 2017).

To ensure that outliers did not affect our results, we preregistered that we would rerun all our analyses while eliminating influential data points identified via analyses of Cook’s distance. We defined an outlier as any observation with a Cook’s distance value greater than three times the mean (this particular criterion was not preregistered). Removing outliers did not change the direction nor significance of the results and therefore we did not exclude any outliers. We also conducted additional checks (not preregistered) where we analyzed the data with overly negative memories and old memories removed. We checked whether negatively valenced memories affected our results due to previous research highlighting how negative emotion affects memory in unique ways (e.g., Bisby et al., 2018). We checked whether old memories affected our results because memory age is also a factor that affects recall (e.g., Hardt et al., 2013). For findings of these analyses, see Table S1 in the Supplementary Material.

#### Profile analysis

In addition to our preregistered analyses, we conducted an analysis of our three memory construction variables (semantic reliance, single-episode reliance, and mixed-episode reliance) using exploratory latent profile analysis to examine whether there were distinct types (or profiles) of repeated-event memories. Latent profile analysis is a categorical latent variable modeling approach that uses a set of continuous variables in an attempt to identify two or more latent subgroups within a broader sample, where each subgroup has a distinct pattern of scores across the set of variables used (for a guide to latent profile analysis, see Spurk et al., 2020). For example, one profile might contain medium scores on all variables; another profile might have high scores on one variable, medium scores on the second variable, and low scores on the third variable; and yet another profile might have high scores on two variables and low scores on the third variable. Thus, we sought to identify repeated-event memory profiles with distinct patterns of scores across our three memory reliance variables.

Nylund et al. (2007) recommends a sample size of 500 subjects to have confidence in the resulting profiles. While Study 2 met this threshold at the memory level, neither of our studies met this threshold at the subject level. Therefore, we report results of profile analyses per study in the main manuscript and results of a cumulative profile analysis using data from both study samples in the Supplementary Material. A holistic consideration of all model indices in the cumulative profile analysis supported the same model type with the same number of profiles reported for Study 1 and Study 2.

The process of determining the best-fitting profile solution relies primarily on the use of quantitative information-criterion statistics and secondarily on principled qualitative judgment on the part of the researchers to determine whether an additional profile increases informativeness. In other words, all profiles should have a distinct shape (see Spurk et al., 2020). To conduct a profile analysis on our memory reliance variables, we used the *mclust* package (Scrucca et al., 2016). The process of our model selection was as follows: First, we looked at the Bayesian information criterion values (Schwarz, 1978) of all the different model types offered in *mclust* from one to nine profiles. We then selected the top performing model for each number of profiles, until successive gains in BIC values taper off, which indicates that gains in model fit are not sufficient to compensate for the penalty term added for decreases in parsimony. We honed in on the best model from this set of candidates by evaluating integrated complete-data likelihood criterion, which adds a penalty to the Bayesian information criterion through an entropy term that measures cluster (i.e., profile) overlap (Biernacki et al., 2000), comparing the relative model fit of neighboring profile models (i.e., models of the same type but with a different number of profiles) using bootstrapped likelihood ratio tests (McLachlan, 1987), and qualitatively judging whether profiles in larger models offered additional explanatory value by examining whether each profile had a distinct shape. We prioritized parsimony in our models, and also rejected models that contained profiles with < 5% of the sample (i.e., fewer than 15 memories in Study 1 and 63 memories in Study 2; Nylund et al., 2007).

For the resulting memory profiles in the model we selected, we conducted follow-up mixed-effect model analyses assessing differences in similarity, arousal, frequency, and vividness between the profiles using the *lme4* (Bates et al., 2015) and *lmerTest* packages (Kuznetsova et al., 2017). We specified the maximal model for these analyses based on guidelines from Barr et al. (2013): Specifically, we began by including a random effect term for participants and a random slope term for profile, and when the resulting models did not converge we eliminated the random slope. We used dummy coding to compare profiles in these models, but we ran each analysis twice while changing the reference group to ensure we made every comparison. We report unstandardized regression coefficients, with *p* values calculated using Satterthwaite’s (1946) method of estimating approximate degrees of freedom. We report 95% confidence intervals in square brackets throughout the Results section.

#### Narrative hypotheses

In our first preregistration, we formulated two narrative hypotheses that were conceptually related but not identical to our self-report hypotheses, which we planned to analyze with an automated scorer of the Autobiographical Interview (Van Genugten & Schacter, 2024). However, in analyzing the data for these hypotheses we determined that the automated scoring tool we used to evaluate semantic and episodic content in the memory reports may not be appropriate for the narratives in our study, as the training data for the tool did not include repeated-event memories. We concluded that a thorough investigation of these hypotheses would require a separate study using different tools, including human scoring, which would go beyond the present paper. In the interest of adhering to the analysis plan outlined in our first preregistration, we have elected to report the method and results we used for these hypotheses in our Supplementary Material.

## Results

We report results of the analyses for both studies side by side throughout our Results instead of sequentially, as Study 2 was a direct replication of Study 1. In line with the main aims of this study, following the description of phenomenological ratings of the collected memories, we report repeated-measures correlation analyses where we investigate whether similarity is associated with where repeated events fall on the semantic–episodic continuum. Next, we describe results of exploratory profile analyses, where we investigate whether there are different types of repeated-event memories according to their relative reliance on semantic memory, a single episode, and a mix of episodes. We then follow-up with analyses investigating whether the profiles are associated with differences in similarity, as well as other variables that we theorized might be intuitively related to the profiles that emerged in our exploratory analyses (including vividness, frequency, and emotional arousal). Additional analyses can be found in our Supplementary Material, including 1) checks and follow-up exploratory analyses related to our primary hypotheses; 2) supplementary results for our latent profile analyses; 3) preliminary results for our preregistered narrative hypotheses; and 4) descriptive results for our content analysis of memory narratives.

### Description of repeated-event memories

In Study 1, we collected 291 repeated-event memories. On average, these events occurred 3.36 times per week in the last month (*SD* = 3.65, min 0, max = 30; 0 means that the event occurred less than once a week in the last month). The mean emotional valence of these memories, measured on a scale from − 5 to 5, was slightly positive (*M* = 1.49, *SD* = 1.99, min =  − 4.9, max = 5.0), with 22 events rated as negative (emotional valence rating lower than − 1), 111 rated as neutral (emotional valence of − 1 to 1), and 158 rated as positive (emotional valence greater than 1).

In Study 2, we collected 1,257 repeated-event memories. On average, these events occurred 3.12 times per week in the last month (*SD* = 3.90, min = 0, max = 60). Similar to Study 1, the mean emotional valence was slightly positive (*M* = 1.59, *SD* = 2.16, min =  − 5.0, max = 5.0), with 125 events rated as negative, 401 rated as neutral, and 731 rated as positive. Other phenomenological ratings for the repeated-event memories in our studies are presented in Table 1. In both studies, we also checked whether participants reported memories that had occurred at least once in the year the study was run (2022 for Study 1 and 2023 for Study 2), and we compared memories that did not meet the criterion with memories that did. We did not find any substantial reason to exclude older memories from our analyses (see supplementary Material for more details).

**Table 1:** Phenomenological ratings (0–10 scale) in Study 1 and Study 2

Variable	Study 1		Study 2	
	*M*	*SD*	*M*	*SD*
Vividness	7.57	2.02	7.77	1.96
Visual detail	7.87	2.03	8.03	1.92
Other sensory detail	6.12	2.68	6.09	2.83
Arousal	5.30	2.54	5.54	2.62
Personal relevance	6.68	2.60	6.54	2.61

Scales were labeled “Not at all” at the low extreme (0), “Moderately” at the middle point (5), and “Very/A lot” at the high extreme (10). Arousal ratings were labeled at the low extreme (0) “Very calm” and at the high extreme (10) “Very aroused.”

### Self-report analyses

We used bootstrapped repeated measures correlations to examine our preregistered self-report hypotheses as well as a series of exploratory correlations between our similarity and memory reliance variables. Descriptive statistics for these variables are listed in Table 2.

**Table 2:** Similarity and memory reliance ratings in Study 1 and Study 2

Variable	Study 1		Study 2	
	*M*	*SD*	*M*	*SD*
Overall similarity	6.65	2.32	7.08	2.31
Similarity of place	8.05	2.61	8.38	2.42
Similarity of people	7.19	2.94	7.51	2.82
Semantic reliance	6.91	2.39	7.01	2.26
Single episode reliance	4.81	2.92	5.01	2.78
Mixed episodes reliance	6.45	2.56	6.75	2.45

The low extreme (0) and high extreme (10) verbal anchors for the similarity variables were as follows: For overall similarity “Very different” to “Very similar”; for similarity of place, “Always a different place” to “Always the same place”; for similarity of people, “Always different people” to “Always the same people.” The three memory reliance variables were all rated on the same scale from “Not at all” at the low extreme (0), “Moderately” at the middle point (5), and “A lot” at the high extreme (10)

#### Semantic hypothesis

**Fig. 1:**
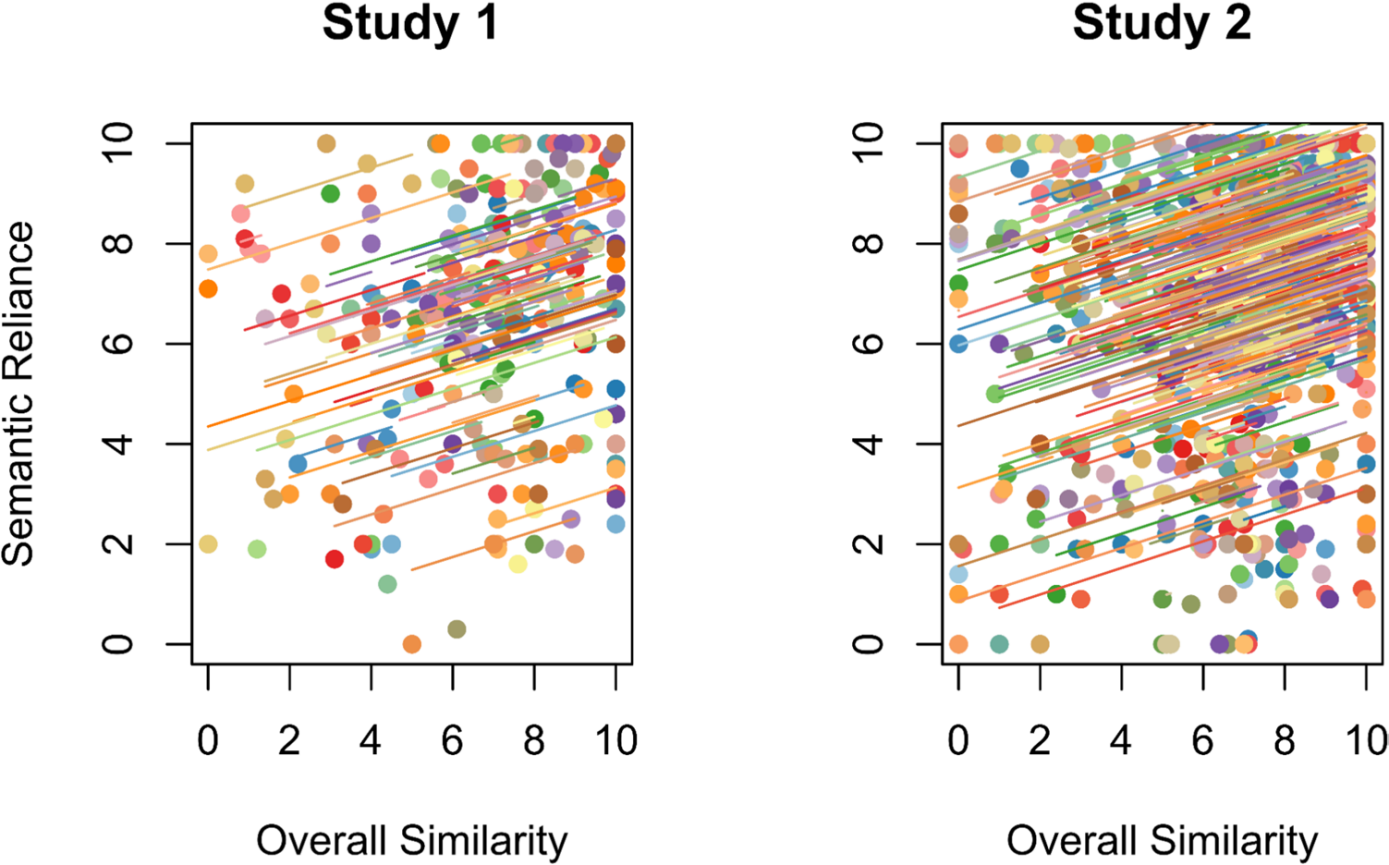
Repeated measures correlations between overall similarity and semantic reliance (H1) in Study 1 and Study 2. *Note.* Data points correspond to individual memories, colors correspond to different participants, and the parallel lines represent the common regression slope with varying intercepts for each participant. (Color figure online)

We found a significant positive correlation between self-reported overall repeated-event similarity and reliance on semantic memory, *r*_*rm*_(193) = 0.23, [0.11, 0.35], *p* = 0.001. We replicated this effect in Study 2, *r*_*rm*_(837) = 0.23, [0.16, 0.30], *p* < 0.001 (Fig. 1). These results are in line with H1 described in both of our preregistrations.

#### Episodic hypothesis

**Fig. 2:**
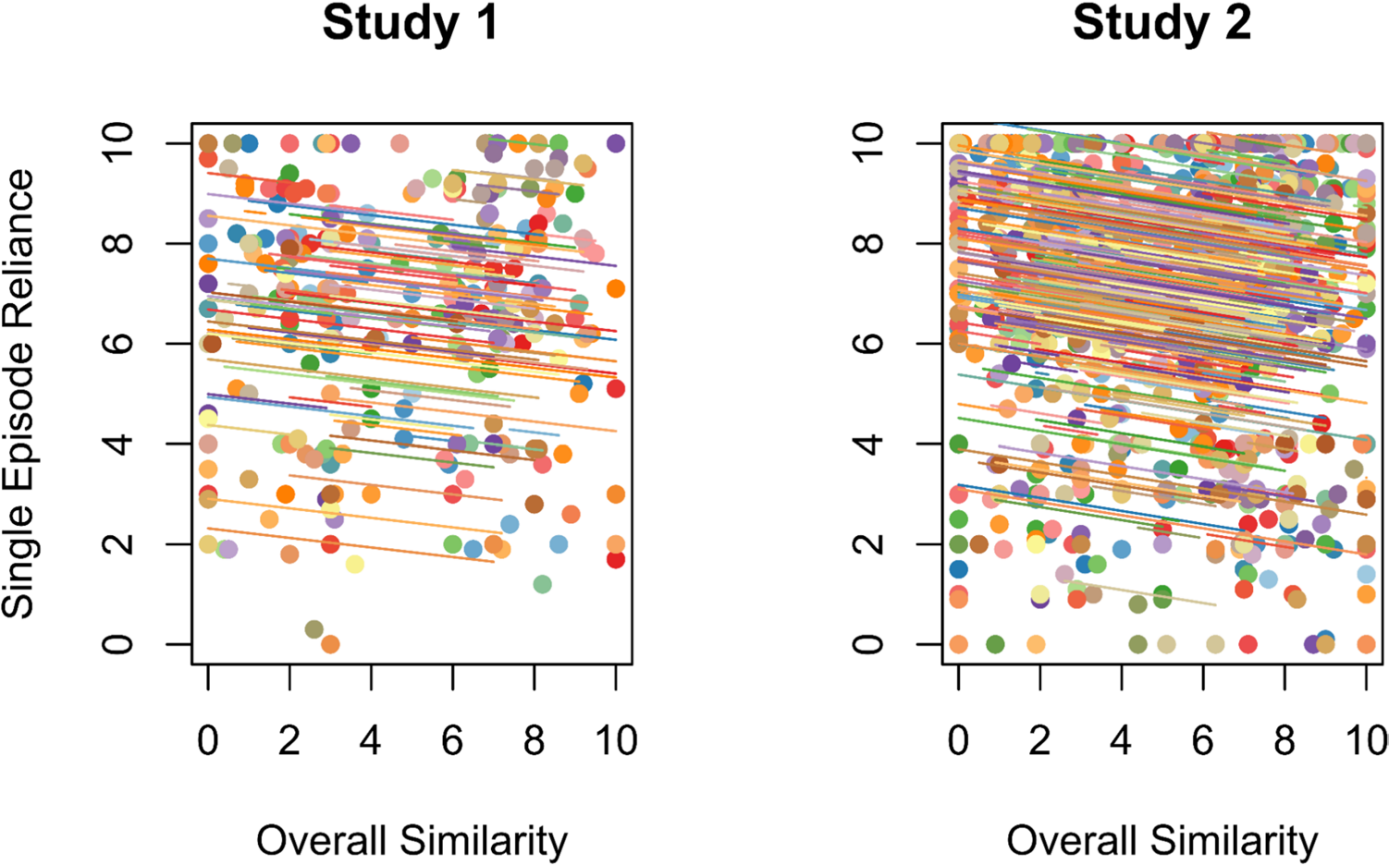
Repeated measures correlations between overall similarity and single episode reliance (H2) in Study 1 and Study 2. *Note.* Data points correspond with individual memories, colors correspond to different participants, and the parallel lines represent the common regression slope with varying intercepts for each participant. (Color figure online)

We expected to find a negative correlation between self-reported overall repeated-event similarity and reliance on a single episode (H2). In Study 1, the effect was not significant, *r*_*rm*_(193) =  − 0.11, [− 0.26, 0.03], *p* = 0.114, and the effect size was smaller than we anticipated based on our pilot sample (expected *r*_*rm*_ =  − 0.21). To determine if the lack of a significant effect was due to low statistical power, we used the episodic hypothesis data from Study 1 to conduct a power simulation for Study 2. In Study 2 we had adequate power to detect the smaller effect size observed for H2 in Study 1 (see Study 2 Method), and indeed we found a small but significant negative correlation between overall similarity and reliance on a single episode, *r*_*rm*_(837) =  − 0.14, [− 0.21, − 0.07], *p* < 0.001 (Fig. 2), in line with H2 in our preregistrations.

### Profile analysis

In accordance with our aim to investigate the diversity of repeated-event memories—and more specifically whether all repeated-event memories are the same in their relative use of semantic memory and episodic memory—we conducted a profile on our three memory reliance variables (semantic reliance, single episode reliance, and mixed episodes reliance) in both of our studies.

#### Study 1

**Fig. 3:**
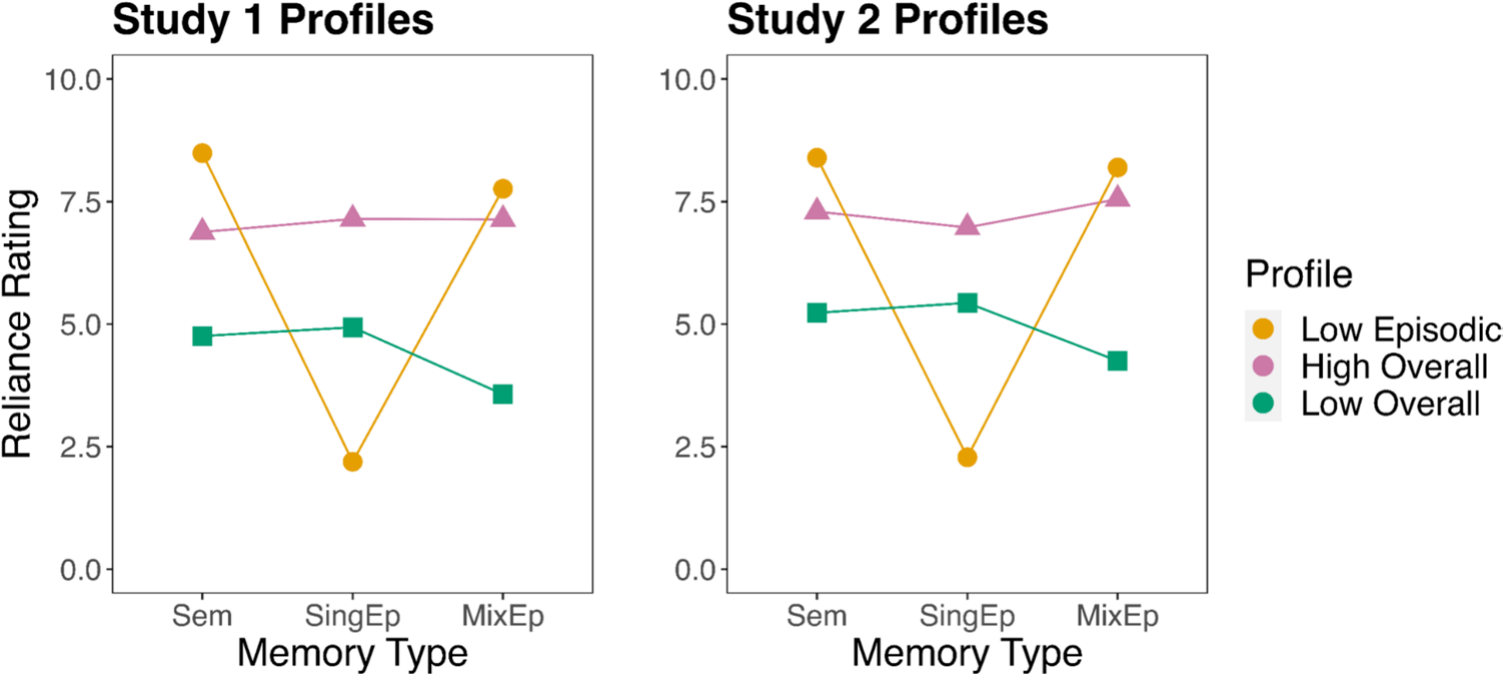
Study 1 and Study 2 mean memory reliance ratings for each profile in the three profile model. *Note*. Sem = semantic reliance. SingEp = single-episode reliance. MixEp = mixed-episodes reliance. (Color figure online)

In our profile analysis for Study 1, we initially selected one two-profile model, one three-profile model, one four-profile model, and one five-profile model for further evaluation based on their Bayesian information criterion (BIC) values (see Fig. S3 in the Supplementary Material). After evaluating entropy (using integrated complete-data likelihood criterion; see Fig. S4) and gains in model fit amongst neighboring models using bootstrapped likelihood ratio tests, we compared the shape of the profiles across models. Based on these analyses, we determined that the best model was the three-profile model. This model had substantially higher model fit than the two-profile model based on bootstrapped likelihood-ratio tests (see Supplementary Material) and the four- and five-profile models contained small profiles (< 5% of the sample size) that were essentially variations of profiles in the three-profile model. We named the three memory profiles according to their shape: The low episodic reliance profile (*n* = 104 memories) was high on semantic and mixed-episodes reliance but low on single-episode reliance; the high-overall-reliance profile (*n* = 113 memories) was high on all three memory reliance variables; and the low-overall-reliance profile (*n* = 74 memories) was low on all three variables. See the Supplementary Material for a full description of our profile analysis for Study 1 (also see Fig. 3).

#### Study 2

We initially selected one two-profile, one three-profile, and one five-profile model for further analysis based on their BIC values (see Fig. S8 in the Supplementary Material). After evaluating entropy (see Fig. S9), gains in model fit between neighboring models, and then comparing the shape of the profiles in each of the models, we determined that the two best models were the three-profile model and the five-profile model: Both had a substantially higher model fit than the two-profile model, while the best four-profile model had lower BIC values than the best three-profile model. Deciding between the three-profile model and five-profile model was more difficult. The three-profile model was more parsimonious (based on our qualitative analysis) and provided a direct replication of the results of Study 1. The five-profile model demonstrated superior model fit and contained a novel but small profile (*n* = 40 memories; i.e., < 5% of memories in the sample), but also had an additional relatively redundant profile similar to one in the three-profile model. Ultimately, we felt more confident in the three-profile solution as it still demonstrated strong model fit. We labeled the memory profiles in the same way as Study 1: low-episodic reliance (*n* = 400 memories), high-overall reliance (*n* = 473 memories), and low-overall reliance (*n* = 384 memories). See the Supplementary Material for a full description of our profile analysis for Study 2.

### Mixed-effect model analyses

To examine whether the abovementioned memory profiles systematically differed from each other on overall similarity, similarity of place, similarity of people, arousal, frequency, and vividness, we conducted a series of exploratory analyses using mixed-effect models (see Table 3 for descriptive statistics). We examined similarity because understanding the role of similarity and some of its facets in repeated-event memory was a core focus of the study. We examined arousal, frequency, and vividness post hoc as we believed there might provide insight into differences among the memories forming the profiles we identified. All analyses were dummy coded, with the low-episodic-reliance profile as the main reference group, though we also report comparisons between the high-overall-reliance and low-overall-reliance profiles, with the high-overall-reliance profile as the reference group. See Table S6 in the Supplementary Material for regression statistics for all of the mixed-effect models we ran in Study 1 and Study 2.

**Table 3:** Means and standard deviations of each profile across five variables

Study	Low episodic		High overall		Low overall	
	*M*	*SD*	*M*	*SD*	*M*	*SD*
Overall similarity						
1	6.72	2.30	6.89	2.12	6.16	2.57
2	7.35	2.09	7.01	2.17	6.89	2.65
Similarity of place						
1	8.73	2.18	7.85	2.61	7.38	2.96
2	8.80	2.10	8.25	2.33	8.10	2.75
Similarity of people						
1	7.58	2.76	7.33	2.75	6.34	3.38
2	7.73	2.83	7.59	2.65	7.17	3.01
Arousal						
1	4.97	2.52	5.97	2.42	4.73	2.56
2	5.33	2.68	5.97	2.44	5.24	2.71
Frequency						
1	3.21	3.22	3.04	2.22	4.09	5.49
2	3.09	3.95	3.06	3.89	3.23	3.87
Vividness						
1	7.84	1.90	7.86	1.70	6.77	2.40
2	7.73	2.01	7.87	1.77	7.71	2.13

The low extreme (0) and high extreme (10) verbal anchors for the similarity variables were as follows: For overall similarity “Very different” to “Very similar”; for similarity of place, “Always a different place” to “Always the same place”; for similarity of people, “Always different people” to “Always the same people.” Arousal ratings were labeled at the low extreme (0) “Very calm” and at the high extreme (10) “Very aroused.” Frequency values were open-ended numeric responses indicating how frequently a particular repeated event happened in a participant’s life in the past month in terms of times per week. Vividness was labeled “Not at all” at the low extreme (0), “Moderately” at the middle point (5), and “Very/A lot” at the high extreme (10)

#### Overall similarity

In Study 1, we found no significant differences in overall similarity ratings when comparing the low-episodic-reliance profile with the high- and low-overall-reliance profiles: When compared with the high-overall-reliance profile, *b* = 0.12, [− 0.51, 0.74], *t* = 0.36, *p* = 0.718, and when compared with the low-overall-reliance profile, *b* =  − 0.64, [− 1.34, 0.06], *t* =  − 1.79, *p* = 0.075. The high-overall-reliance profile was associated with higher similarity ratings than the low-overall-reliance profile, *b* =  − 0.76, [− 1.45, − 0.06], *t* =  − 2.13, *p* = 0.034.

In Study 2, we found that the low-episodic-reliance profile was associated with higher overall similarity ratings than both the high-overall-reliance profile, *b* =  − 0.36, [− 0.68, − 0.05], *t* =  − 2.24, *p* = 0.025; and the low-overall-reliance profile, *b* =  − 0.52, [− 0.85, − 0.19], *t* =  − 3.06, *p* = 0.002. There was no significant difference between the high-overall profile and low-overall profile (*p* = 0.332). Thus, the pattern of effects we observed for overall similarity were largely inconsistent across the two studies, so these results should be interpreted cautiously.

#### Similarity of place

In both studies, memories in the low-episodic-reliance profile received higher similarity of place ratings than memories in both other profiles: When compared with the high-overall-reliance profile, in Study 1, *b* =  − 0.89, [− 1.57, − 0.20], *t* =  − 2.53, *p* = 0.012; and in Study 2, *b* =  − 0.53, [− 0.85, − 0.20], *t* =  − 3.18, *p* = 0.002; and when compared with the low-overall-reliance profile, in Study 1 *b* =  − 1.37, [− 2.14, − 0.60], *t* =  − 3.49, *p* < 0.001; and in Study 2, *b* =  − 0.71, [− 1.05, − 0.37], *t* =  − 4.09, *p* < 0.001. There were no significant differences between the high- and low-overall-reliance profiles in either study (*p* values ≥ 0.213).

#### Similarity of people

Memories were excluded from the similarity of people analysis in Study 1 (*n* = 45 excluded) and Study 2 (*n* = 194 excluded) if a participant indicated that they experienced the repeated event alone. In both studies, there were no significant difference in similarity of people ratings when comparing the low-episodic-reliance profile (Study 1: *n* = 89; Study 2: *n* = 340) with the high-overall-reliance profile (Study 1: *n* = 98, *p* = 0.516; Study 2: *n* = 413, *p* = 0.545). However, similarity of people ratings were higher in the low-episodic-reliance profile compared with the low-overall-reliance profile in both studies: Study 1, *n* = 59, *b* =  − 1.24, [− 2.21, − 0.27], *t* = −2.50, *p* = 0.013; Study 2, *n* = 310, *b* =  − 0.52, [− 0.96, − 0.08], *t* =  − 2.33, *p* = 0.020. There were no significant differences between the high-overall-reliance profile and low-overall-reliance profile in either study (*p* values ≥ 0.052).

#### Arousal

In both studies, memories in the high-overall-reliance profile received higher arousal ratings than memories in both other profiles: When compared with the low-episodic-reliance profile, in Study 1, *b* = 0.94, [0.26, 1.62], *t* = 2.74, *p* = 0.007; and in Study 2, *b* = 0.56, [0.20, 0.91], *t* = 3.06, *p* = 0.002; and when compared with the low-overall-reliance profile, in Study 1 *b* =  − 1.12, [− 1.90, − 0.36], *t* =  − 2.95, *p* = 0.003; and in Study 2, *b* =  − 0.60, [− 0.96, − 0.24], *t* =  − 3.32, *p* < 0.001. There were no significant differences between the low-episodic-reliance and low-overall-reliance profiles in either study (*p* values ≥ 0.638).

#### Frequency

In both studies, we did not observe significant differences between memory profiles (Study 1: *p* values ≥ 0.099; Study 2: *p* values ≥ 0.517) when examining the frequency of the repeated event per week within the last month.

#### Vividness

In Study 1, we found no significant difference when comparing the low-episodic-reliance profile to the high-overall-reliance profile (*p* = 0.732). However, we found that the low-episodic-reliance profile was significantly higher in vividness than the low-overall-reliance profile, *b* =  − 0.64, [− 1.25, − 0.04], *t* =  − 2.13, *p* = 0.034. The high-overall-reliance profile was also significantly higher in vividness than the low-overall-reliance profile; *b* =  − 0.73, [− 1.33, − 0.13], *t* =  − 2.44, *p* = 0.012. In Study 2, we did not observe significant differences in vividness between memory profiles (*p* values ≥ 0.138). Since this effect did not replicate in the larger sample, the findings in Study 1 were likely spurious.

## Discussion

The present study is the first to apply the continuum perspective to investigate whether similarity is associated with episodic and semantic memory in repeated-event recall. Across two naturalistic samples, we found evidence in support of two preregistered predictions: Higher self-reported similarity between repeated-event instances was associated with 1) greater self-reported reliance on semantic memory and 2) less self-reported reliance on a single episode, although the latter relationship was only significant in Study 2, potentially because Study 1 could have been underpowered to detect this effect.

Why was high similarity associated with greater reliance on semantic memory? It has been argued that stronger repeated-event memory scripts are formed when instances are more similar. This may facilitate attentiveness to deviations from the event’s normal course (e.g., Farrar & Goodman, 1992), but interferes with the ability to correctly attribute information to the instance in which it was acquired (i.e., source monitoring; Danby et al., 2019; Johnson et al., 1993; Lindsay, 2008, 2014). In our study it is plausible that higher similarity repeated events were associated with stronger scripts, which may have interfered with the ability of participants to clearly parse out a single episode. Conversely, lower similarity repeated events may have been associated with less detailed scripts and thus reduced reliance on semantic memory. This interpretation, and our results more broadly, nest well in the extant repeated-event memory literature (e.g., Danby et al., 2019).

Critically, our study differs from prior research in that we examined retrieval dynamics of episodic content across different levels of similarity. We were particularly interested in whether lower similarity is associated with increased reliance on a single episode (i.e., a single instance of a repeated event) during recall, and in Study 2 we found evidence for this association. One feature of our study design that differs from previous repeated-event research is that we did not explicitly ask participants to remember single instances during recall. Rather, our results pertaining to single-episode reliance can be interpreted as capturing the degree to which participants naturally represented a single instance (i.e., an episode) of a repeated event during recall, without prompting. A script perspective can help explain why instances of low-similarity repeated events are less integrated within a repeated-event script at encoding, but at retrieval, the script perspective emphasizes the tendency to recall fixed details (that form the script) as opposed to details that are variable across instances or that are unique to a single episode. Following this, the script perspective offers limited insight as to why single instances seem to spontaneously appear to participants during recall of some low-similarity repeated events.

It is also worth highlighting that the majority of repeated-event memories in our study demonstrated a moderate to high degree of reliance on a mix of episodes, which was positively correlated with reliance on semantic memory and negatively correlated with reliance on single episode (see Fig. S2 in the Supplementary Material). Mixed-episode reliance had higher mean ratings than single-episode reliance, which indicates that participants often pictured episodic information from several episodes when recalling repeated events. These findings raise an intriguing question regarding the interplay of semantic and episodic memory as it pertains to the phenomenology of repeated-event scripts: Are scripts simply known factually, or are they accompanied by episodic content, such as a single un-amalgamated episode (i.e., a single instance), a single amalgamated episode (e.g., a “repisode”; see Neisser, 1981), or remembrance of a series of distinct episodes/instances in sequence?

Results from our exploratory profile analyses lend some preliminary evidence that the phenomenology of repeated-event recall might vary depending on the repeated-event memory. In both studies, we identified three unique profiles of repeated-event memories that differed in their relative use of semantic and episodic memory: A low-episodic-reliance profile where repeated-event memories were rated high on semantic- and mixed-episodes reliance but low on single-episode reliance, and two profiles of repeated-event memories that were balanced on all three memory-reliance variables (dubbed high-overall reliance and low-overall reliance, respectively). These results broadly indicate that reliance on episodic and semantic memory is not always mutually exclusive; that is, memories are not binary in their representational format. Given that events unfold in time in our memories, it is possible that participants held multiple semidistinct representations that oscillated over the course of remembering. The low-episodic-reliance profile suggests that some repeated events tend to be devoid of drawing on single “pure” episodes, but instead rely on abstracted or accumulated knowledge, whereas for high-overall-reliance memories, all forms of representational content are used to construct the event. The low-overall-reliance profile is somewhat surprising and may represent a general difficulty in forming a coherent memory representation, though this profile did not reliably differ from its counterparts in vividness.

Our follow-up analyses investigating whether there were common features amongst repeated-event memories clustered within each profile provided some further insight into the idiosyncratic retrieval dynamics. When considering different facets of similarity (i.e., overall, place, people), place similarity produced the clearest pattern: Memories within the low-episodic-reliance profile yielded higher similarity of place ratings relative to the other two profiles, with no differences between memories within the latter profiles. This finding suggests that repeated events occurring in one place (or few places) draw less on a single episode/instance. This is consistent with other work suggesting that variability of place is a key factor that is associated with the recollection of distinct instances of a repeated event (MacLean et al., 2018; also see Robin et al., 2018).

We also found that arousal differentiated memory profiles: Repeated-event memories with high levels of arousal tended to cluster within the high overall reliance profile. Emotional arousal is known to affect memories in complex ways. For example, it can enhance memory encoding and consolidation for individual items while often simultaneously impairing association memory, including binding items to their context (Fujiwara et al., 2021; Madan et al., 2012; Mather, 2007; Schümann & Sommer, 2018; Talmi & Palombo, 2025). Judging causality for this effect is difficult because it is possible that experiencing multiple representations at recall led to an increased sense of intensity. Future research is needed to understand the direction of causality and the role of arousal in repeated-event memory more generally (see Price & Connolly, 2008).

### Theoretical implications

The notion that repeated events are an intermediate form of memory represents an important paradigm shift in memory research (see Greenberg & Verfaellie, 2010; Renoult et al., 2012; Tanguay et al., 2023) though the seeds for this idea were presented even by Tulving (1972). While it has been acknowledged that there is a degree of episodicity in repeated events (e.g., Rubin & Umanath, 2015), there is a dearth of research on repeated events from this perspective. By showing that similarity is associated with where repeated-event memories fall along the semantic–episodic continuum, our study serves as a bridge between the applied/developmental repeated-events literature, where similarity was first highlighted as a potential factor affecting the ability to recall instances of repeated events (Danby et al., 2019), and the autobiographical memory literature, where the continuum perspective was developed and refined (e.g., Renoult et al., 2012).

Our findings also suggest that adopting a continuum perspective may provide additional insight into repeated-event memory above and beyond a semantic memory (e.g., script) perspective, and raise a variety of questions concerning the interplay of episodic and semantic memory during repeated-event recall, especially regarding the phenomenology of repeated-event scripts (i.e., the extent to which they are known factually or imagined as events). A continuum perspective may, for example, enable investigating differences in the ability to recall instances across different types (i.e., profiles) of repeated events, and could further integrate insights regarding different phenomenological aspects of memory for this purpose. Furthermore, our follow-up analyses investigating factors associated with the different memory profiles highlights some key experimental manipulations which could enhance the generalizability of repeated-event memory research. Specifically, our results suggest that experimental paradigms that do not vary the setting of stimuli or the place where stimuli are presented may bias participants towards recalling one type of repeated event (i.e., repeated events most similar to those in our low-episodic-reliance profile). Furthermore, paradigms which do not vary the emotional arousal of repeated events may fail to capture all types of repeated-event memories that can arise during recall.

We have primarily focused on comparing a continuum perspective with a script perspective, but it is worth considering the implications our results have for other theories of memory. First, it is worth stating that, in conjunction with our findings implicating similarity of place in repeated-event memory, the fact that all types of repeated-event memories described in this study demonstrated at least some reliance on different forms of episodic content (either a single episode or a mix of episodes) lends support to Rubin and Umanath’s (2015) proposed reconceptualization of episodic memory as event memory, where an event is rooted in scene construction and encompasses both single and repeated events.

Our work highlights the value of conceptualizing declarative memories as products of functional interactions between semantic and episodic memory. In this respect, our findings are consistent with neuroimaging work indicating that memories ranging from semantic, personal semantics (including repeated events), to unique episodes activate a common neural network with unique signatures for each type of memory (Addis et al., 2004; Levine et al., 2004; Svoboda et al., 2006; Tanguay et al., 2023). What is perhaps most clear from our investigation is that, especially in event studies, episodic and semantic memory ought to be examined together, rather than apart, regardless of whether retrieval interactions are a product of one, two, or more memory systems.

It is important to consider how our data fit with recent multidimensional accounts of memory (Addis & Szpunar, 2024; also see Rubin, 2022). Some have argued that distinguishing semantic and episodic memory by placing them on a continuum with two “pure” versions at either end, may risk conflating distinct contributions from several dimensions, including temporal specificity (specific vs. general), content (perceptual versus conceptual), and self-reference (idiosyncratic vs. shared; Addis & Szpunar, 2024). Indeed, results from our profile analysis do highlight this limitation: We queried participants about their reliance on both a single episode and a mixture of episodes, and our results highlight that these variables did not consistently move together, suggesting there are multiple ways that episodic-esque content can manifest in memory. It is conceivable that single-episode reliance captured more temporally specific and less conceptual content than mixed-episodes reliance, as mixing episodes is inherently less temporally specific and may entail the conceptual understanding that the episodic content of one’s memory draws from multiple instances of a repeated event. A multidimensional perspective may have elucidated such representational dynamics, and this is certainly an appealing avenue for future research to explore.

### Practical implications

The findings of this study—and research on repeated events more generally—have applied value. Research shows that memory reports of instances of repeated events are perceived as less credible than reports of single events (Weinsheimer et al., 2017). Our findings suggest avenues for further research in credibility evaluations. For example, the continuum perspective might serve as an intuitive framework to help jurors grasp the challenges of recalling instances of repeated events with varying similarity. Indeed, the initial impetus for studying memories of repeated events in children was to understand the veracity of children’s eyewitness testimony, often in cases of repeated abuse (Brubacher et al., 2014). Adult research on repeated events is equally valuable (Deck & Paterson, 2020). Along similar lines, investigations of workplace accidents (e.g., an injury sustained at work) rely on a witness’s ability to recount a distinct work day—an (often) highly similar repeated event (see Kelloway et al., 2004). Our findings suggest that similarity of repeated events could be a crucial part of understanding a person’s ability to provide specific testimony about a particular instance. For example, it may be relevant to inquire about whether events pertaining to a particular legal case or workplace investigation occurred in one place or in multiple places, as that is one key source of variability regarding how repeated events are remembered. Still, it should be noted that our study does not provide insight pertaining to the accuracy of recall under different conditions of similarity.

Research on repeated events is clinically relevant. Memories of repeated events are often examined in the context of trauma or abuse (Levy-Gigi et al., 2014; Woiwod & Connolly, 2017; also see Giourou et al., 2018). Maladaptive schemas have also been shown to play a role in mood and anxiety disorders (Renner et al., 2012; Tariq et al., 2021). Given that repeated events play a key role in schema (e.g., script) formation and alteration (see Gilboa & Marlatte, 2017; Rubínová et al., 2021), studying them could yield valuable insights into various mental health conditions.

### Limitations

Given that our study was limited to recruitment from participant pools at Western universities, our findings are not representative of the general population. Cross-generational and cross-cultural examinations of our core findings are a crucial follow-up.

Our study is also limited to the use of self-report, which could also raise concerns about generalizability. Although we gave participants detailed explanations and comprehension checks, it is possible that some participants did not understand some memory concepts in the way we hoped or made inaccurate judgments about which forms of memory they were using. Relatedly, it is possible that the language we used in our instructions—such as asking participants if they “pictured” episodes in their memory—biased participants to conflate episodic memory with mental imagery, despite the fact that mental imagery can be present in both semantic and episodic forms of memory (see Renoult et al., 2012). Though certain design choices can give rise to demand characteristics, we did not find any participants who correctly guessed the purpose of the study, so we do not have any evidence that demand characteristics were present: Many participants thought the study was about the effects of vividness or emotion/mood on memory.

At a broader level, asking participants to report on aspects of their memories that they do not normally reflect upon may have affected their memories in unpredictable ways. For example, because we asked participants to report on visual/sensory imagery, they may have developed the visual/sensory imagery of their memories more than they otherwise would have. It is also unclear whether the order in which we presented the questions contributed to the pattern of results. In part as a means of limiting the amount of noise in our data, we followed a common practice in research of asking questions in a fixed as opposed to random order. It would be potentially beneficial for future research—particularly experimental work where the noise inherent in naturalistic sampling is mitigated—to randomize the order of questions.

As a final note of caution, the magnitude of our correlations were generally small to modest, which was perhaps related to the noise inherent in our naturalistic approach to sampling memories, but could also suggest unexplained variance in our data. Yet, with the exception of the association between single episode reliance and similarity, we replicated all the effects we interpret across both studies, which bolsters confidence in our results. Furthermore, the associations we observed may still have practical significance given the pervasiveness of repeated-event memory in everyday experience.

### Future directions

Our study could inform future investigations in both experimental and applied domains focusing on the role of similarity in various forms of memory on the full spectrum of the semantic–episodic continuum. One follow-up that we believe will be a fruitful avenue for repeated-event memory research is inquiring further into what participants mean when they indicate recalling a mix of episodes: Are they recalling multiple episodes/instances in sequence, a single amalgamated event memory which draws on multiple instances, or do they mean something else entirely?

Likewise, conducting a more thorough investigation into the various facets of similarity could yield additional, novel insight. In our study we asked participants about two aspects of similarity: similarity of place and similarity of people. Yet there are a variety of aspects of similarity that we did not probe, such as perceptual, temporal, and emotional similarity, to name a few. Perceptual similarity might be especially important to examine, to elucidate whether the effect of similarity of place is due to the amount of perceptual diversity across repeated events or because place per se is important for memory (see Alme et al., 2014, for rodent research related to this topic). Future research could also examine whether place is a useful memory cue in cases where multiple repeated events occur in the same place (e.g., at home).

Combining our paradigm with narrative research integrating repeated-event scripts or Autobiographical Interview scoring techniques could lead to a deeper appreciation of the diversity of repeated-event memories, particularly if used to further compare the different repeated-event memory profiles observed in our study (see Renoult & Rugg, 2020, for a discussion on Autobiographical Interview scoring for personal semantic forms of memory, including repeated events). Indeed, we initially planned on using an automated scorer of the Autobiographical Interview to investigate the narratives collected in our study, but ultimately decided that a more thorough investigation using hand-scoring techniques specifically tailored for repeated events was warranted.

Future research could also examine whether the repeated events people tend to report vary according to age and culture, and whether “life scripts”—cultural expectations about the normal sequence of life events—are associated with the repeated events people of different ages and cultures report (see, e.g., Berntsen & Bohn, 2009). Congruence or deviation from life scripts could, for example, be associated with how people describe, visualize, and feel about their repeated-event memories.

### Conclusion

Repeated-event memories are integral to our everyday lives but are understudied in the autobiographical memory literature. Our study demonstrates that similarity is associated with where repeated-event memories fall along a continuum from episodic to semantic memory, shining a further spotlight on Tulving’s early sentiment that these two memory processes interact and are not fully separable. More broadly, a continuum perspective–combined with our novel use of profile analysis in repeated-event memory research–enabled us to appreciate the diversity amongst repeated events, suggesting that this form of remembering is nuanced and multifaceted. We hope our study opens new doors for research on this prevalent but often overlooked aspect of human memory.

## Supplementary Information

Below is the link to the electronic supplementary material.

Supplementary file1 (DOCX 5000 kb)

## Data Availability

Open (de-identified) data and materials (Qualtrics survey) for this study are available online (osf.io/hxrc6).

## Footnotes

1 Other newer models and perspectives argue that episodic and semantic forms of mentation are “different outputs from one system that is fundamentally combinatorial and creates transient representations” (Addis & Szpunar, 2024, p. 9), sharing some ideas both with earlier processes views and perspectives that emphasize the interrelated nature of episodic and semantic memory. Some of these models provide important critiques of a continuum perspective (e.g., Addis & Szpunar, 2024).

2 Despite their significant overlap, it is important to note that these different forms of memory also demonstrate some unique neural signatures (e.g., Addis et al., 2004; Levine et al., 2004; Svoboda et al., 2006; Tanguay et al., 2023).

3 Intriguingly, repeated event memories sometimes appear as single episodes but actually represent a series of related episodes. For example, integrating information from several meetings with the same people in the same place into a single cohesive episodic-like memory—a phenomenon dubbed “repisodic memory” by Neisser (1981).

4 A duplicate exclusion stage was implemented due to a technical issue with the Qualtrics survey integration into the UBC Sona system (where students sign up for research studies in return for credit) that led to failure of the automatic credit granting system. Although participants were instructed to contact the researcher to obtain credit, some failed to follow these instructions and instead returned and completed the survey a second time to attempt to get credit. Some used different identifiers, so we added the manual checking step to ensure no duplicate responses with different identifiers were included.

5 The University of Aberdeen Research Ethics Board asked us to add a note in the consent form of our survey telling people to avoid recalling stressful repeated events (the remainder of the consent form and the surveys themselves were identical across locations). This instruction did not impact our results (see Supplementary Material).

## Code availability

The R script used to clean, analyze, and check the results for this study are available online (osf.io/hxrc6).

## Declarations

### Open science and transparency

Open (de-identified) data and materials (R Script and Qualtrics survey) for this study are available online (osf.io/hxrc6). Study 1 and Study 2 were both preregistered and are available on OSF (Study 1: https://osf.io/3avyx; Study 2: https://osf.io/d9ph5).
