# Supplementary material for: Similarity is associated with where repeated-event memories fall on the semantic–episodic continuum
Source: Mem Cognit. 2025 May 20;53(8):2635–54. doi: 10.3758/s13421-025-01729-6 (PMC12696121; doi:10.3758/s13421-025-01729-6)
Supplement: Supplementary file 1 — Supplementary file1 (DOCX 5000 kb) [file 13421_2025_1729_MOESM1_ESM.docx]

# Similarity is Associated with Where Repeated Event Memories Fall on the Semantic-Episodic Continuum: Supplementary Material

## Method

### Survey Instructions and Questions

In the following we provide verbatim survey instructions and questions. Square brackets (“[ ]”) indicating survey flow elements.

[Survey start → participants provide consent and complete our demographics questionnaire]

“In the following questions you will be asked to come up with examples of **repeated** **events** that you experience in **your own life.**
 By **event**, we mean a specific activity that typically lasts **no more** than a few hours.
 By **repeated** we mean that this activity occurs **at least** **once** every two weeks and that it has occurred more than **five** times. [Click]

These events may occur more frequently than every two weeks, but must occur at least once every two weeks.
Later we will ask you to describe your memory of these repeated events in as much detail as possible. [Click]

Examples of repeated events could include:

*'Going to the gym'*

*'Attending lectures for my PSYC 100 course'*

*'Playing a soccer game on the weekend'*

*'Going out for dinner with my partner'*

*'Meeting with my supervisor at work'* [Click]

As you can see, these are specific activities that all occur at least once every two weeks and that would last no longer than a few hours. [Click]

[Question] Would you say that you experience the sort of repeated events described above in your own life?

1. Yes
2. No [Click]

[Comprehension check]: The events I come up with should:

1. Be repeated events that occur at least once every two weeks
2. Be unique, novel events [Click]
   1. [If participants answered b), they were told:]

**Incorrect**.

Please remember that you should list **repeated events** that occur at least once every two weeks. [Click]

Please name **3 repeated events** that you experience in your own life in the spaces below. As a reminder, these events should occur at least once every 2 weeks, and should concern specific activities that last no more than a **few hours.**
Feel free to use the provided examples for inspiration **but try to generate your own events that are relevant to your life**.
Please **DO NOT** use the same repeated event more than once. Each repeated event should be different than the others.
 Just to remind you, some examples of appropriate responses to one of the fill in the blanks below could be:
 *'Going to the gym'
'Attending lectures for my PSYC 100 course'
'Playing a soccer game on the weekend'
'Going out for dinner with my partner'
'Meeting with my supervisor at work'*

Repeated Event 1 [Text Box]

Repeated Event 2 [Text Box]

Repeated Event 3 [Text Box] [Click]

In the following tasks you will be asked to answer questions about the three **repeated events** you named. [Click]

When the instruction is given, please take a few moments to retrieve your memory of the repeated event as **vividly** as possible. After this, you will be asked a series of questions about the repeated event you pictured.
Please press the arrow button to begin. [Click]

Please think about your memory of the repeated event "[RepEv1 [or] RepEv2 [or] RepEv3 ChoiceTextEntryValue]" for 15 seconds. [Click after 15 second timer]

Please answer some questions on the vividness, emotionality, arousal, and personal relevance of “[RepEv1 [or] RepEv2 [or] RepEv3 ChoiceTextEntryValue]”.

[Vividness question] How vivid is your memory of the repeated event?

- [Participants answered with sliding scale from 0.0 (Not vivid at all) to 5.0 (Moderately vivid) to 10.0 (Very vivid)]

[Visual detail question] My memory of this repeated event involves visual details:

- [Participants answered with sliding scale from 0.0 (Not at all) to 5.0 (Moderately) to 10.0 (A lot)]

[Other sensory detail question] My memory of this repeated event involves other sensory details (sounds, smells, and/or tastes):

- [Participants answered with sliding scale from 0.0 (Not at all) to 5.0 (Moderately) to 10.0 (A lot)]

[Emotional valence question] In general, how emotional do you find the repeated event you just imagined?

- [Participants answered with sliding scale from -5.0 (Intensely negative) to 5.0 (Neutral) to 10.0 (Intensely positive)]

[Arousal question] In general, how do you feel when experiencing this repeated event? Note: "aroused" refers to the state of being excited or agitated

- [Participants answered with sliding scale from 0.0 (Very calm) to 10.0 (Very aroused)]

[Personal relevance question] How personally relevant is this repeated event to you? In other words, to what degree is this repeated event personally meaningful to you?

- [Participants answered with sliding scale from 0.0 (Not relevant at all) to 5.0 (Moderately relevant) to 10.0 (Very relevant)] [Click]

[Narrative question] Please take a few moments to describe your memory of “[RepEv1 [or] RepEv2 [or] RepEv3 ChoiceTextEntryValue]” in as much detail as possible. Your response must be at least 150 characters but I encourage you to keep typing until you reach a natural ending point. I want you to describe everything you are remembering so that I can picture what you are picturing. [Click]

[Participants answered the above questions for all three repeated events before proceeding to the next block]

Please answer some questions on the timing and similarity of “[RepEv1 [or] RepEv2 [or] RepEv3 ChoiceTextEntryValue].” [Click]

[Frequency question] On average, how frequently has “[RepEv1 [or] RepEv2 [or] RepEv3 ChoiceTextEntryValue]” happened in your life in the past **month**?

Please provide your answer in terms of times per week. For example, an event that occurs three times a week would be written as "3". An event that occurs once every two weeks would be written as "0.5".

- [Text box, only numeric answers accepted]

[Space between instances question] On average, how many days pass between each time you experience this repeated event?

- [Text box, only numeric answers accepted]

[First time experienced question] When (approximately) was the first time you experienced this repeated event?

If you are unsure about the exact day and/or month of the first occurrence of this repeated event, please enter "00" for each respective field and enter the year of the first occurrence. Please try to be as accurate as possible.

- [Participants answered with three text boxes: one for “Day (dd),” one for “Month (mm},” and one for “Year (yyyy)”]

[Last time experienced question] When was the last time you experienced this repeated event?
If you are unsure about the exact day and/or month of the most recent occurrence of this repeated event, please enter "00" for each respective unknown field and enter the year of the most recent occurrence. Please try to be as accurate as possible.

- [Participants answered with three text boxes: one for “Day (dd),” one for “Month (mm},” and one for “Year (yyyy)”]

[Overall similarity question] How similar is each instance of this repeated event? In other words, is each time you experience this event the same or different?

- [Participants answered with sliding scale from 0.0 (Very different) to 10.0 (Very similar)]

[Similarity of place question] How similar is the setting of this event? In other words, does this event occur at the same place or at different places?

- [Participants answered with sliding scale from 0.0 (Always a different place) to 10.0 (Always the same place]

[Similarity of people question] How similar are the people at each instance of this repeated event? In other words, are there always the same people attending, or are there always different people?

- [Participants answered with a sliding scale from 0.0 (Always different people) to 10.0 (Always the same people). Participants could also check a box provided if they experience the event alone] [Click]

We will now ask you a series of questions about how you construct your memory for “[RepEv1 [or] RepEv2 [or] RepEv3 ChoiceTextEntryValue]” and whether you are picturing episode(s) in your mind or using your acquired knowledge/expectations of the repeated event rather than picturing any episode(s) in particular.

For the purpose of these questions, an **episode** refers to a single memory of a specific event that occurred just once over the course of a few hours (and not longer than a day). In contrast, **acquired knowledge or expectations** refers to general knowledge that could be applied to more than one episode or that may not apply to any episode in particular. [Click]

​​In the context of repeated events, memory of an **episode** would be a recollection of a particular instance of a repeated event. For example: *When I went to the gym last Tuesday, I ran on the treadmill for 3km.*

Conversely, memory of **acquired knowledge or expectations** would include the recollection of general facts that could be applied to several episodes of the repeated event, or to no episodes in particular. For example: *Usually, when I go to the gym I lift weights* (which could be applied to most episodes of this repeated event)*.* Or, another example: *I've learned that doing less reps with more weight is good for building muscle* (which may not be applicable to any episode in particular, but is still relevant to the memory of the repeated event). [Click]

[Comprehension check] In the context of this study, an **episode** refers to:

1. A particular instance of a repeated event
2. General facts that could be applied to several instances of a repeated event
3. Memory of a repeated event as a whole [Click]
   1. [If participants answered b) or c), they were told:]

**Incorrect**.

As a reminder, in the context of this study an **episode** refers to a particular instance of a repeated event. [Click]

[Comprehension check] In the context of this study, **acquired knowledge and expectations** refers to:

1. A particular instance of a repeated event
2. General facts that could be applied to several instances of a repeated event
3. Memory of a repeated event as a whole [Click]
   1. [If participants answered a) or c), they were told:]

**Incorrect**.

As a reminder, in the context of this study **acquired knowledge and expectations** refers to general facts that could be applied to several instances of a repeated event. [Click]

[Single episode reliance question] To what degree do you believe you are utilizing a memory of one **single** episode when constructing your memory of this repeated event? (Reminder: an episode is a single memory of a specific event that occurred just once over the course of a few hours).

- [Participants answered with sliding scale from 0.0 (Not at all) to 5.0 (Moderately) to 10.0 (A lot)] [Click]
- [If participants provided a rating greater than 0 they were asked:]
  - - [Uniqueness follow-up question] ​​Why is this particular episode of this repeated event significant to you? If you don't find this particular episode significant, please select "Other" and type "NA" in the box.
      1. This episode was more emotional than other episodes
      2. This was the first time I experienced this event
      3. This was the most recent time I experienced this event
      4. This episode was somewhat different/distinct from how the event usually goes
      5. Other (please describe): [Text box] [Click]
- [If participants answered d) they were asked:]
  - [Distinct follow-up question] What made this particular episode distinct from how the event usually goes? [Text box] [Click]

[Semantic reliance question] To what degree do you believe you are utilizing your **acquired knowledge or expectations** about how this event **usually** goes, rather than thinking about any specific episode or episodes in particular?

- [Participants answered with sliding scale from 0.0 (Not at all) to 5.0 (Moderately) to 10.0 (A lot)] [Click]

[Mixed episodes reliance question] To what degree do you believe you are **mixing** memories of **several episodes** in this memory. In other words, do you believe you are picturing multiple episodes of this event and, if so, to what degree?

- [Participants answered with sliding scale from 0.0 (Not at all) to 5.0 (Moderately) to 10.0 (A lot)] [Click]

[Other comments question] Do you have any other comments regarding how you're constructing your memory for this repeated event? In other words, do you believe you are drawing your memory from any other sources other than the ones listed above? If your answer is no, please type "NA."

[Participants answered the above questions for all three repeated events, then completed the VVIQ (Marks, 1973), CESD (Radloff, 1977), and shortened STAI (Zsido et al., 2020). After this they were debriefed and the study ended).]”

### Data Cleaning

All data cleaning and data analysis was performed in R Statistical Software (v4.3.1; R Core Team, 2023) using the tidyverse package (Wickham et al., 2019). Data were cleaned by importing the raw Qualtrics data into R and then removing the unnecessary rows and columns. We then filtered the data in order to remove any duplicate identifiers (meaning that only the first response by each participant was kept, regardless of whether it was incomplete or not). This ensured that responses were not biased by participants retaking the survey. After removing duplicate ids, incomplete responses were removed by removing everyone who failed to respond to the last question of the survey. Then all the participants who responded incorrectly to the attention check were removed. After this, we created a variable that summed all the scores from the three comprehension checks (which were coded so that correct answers were given a one and incorrect answers were given a zero). This variable was used to filter the dataset so as to only retain people who had a total comprehension score greater than or equal to 2 (meaning they got at least two out of three of the comprehension checks correct). This was deemed to be a sufficient level of understanding of the study because even when participants got a comprehension check wrong we reminded them of the correct answer. The datasets were then filtered so that only people between the ages of 18 and 35 were retained.

We also implemented two manual checks to detect duplicates. First, we filtered the data such that only identifiers that shared the first four digits were kept, and then two scorers independently compared the responses with similar identifiers to judge whether they were duplicates, based on the demographic information and the memories provided. Across the ten pairs of responses flagged as being potential duplicates, both scorers identified the same four duplicate responses and both judged that the rest of the memories were unlikely to be duplicates (i.e., there was 100% agreement amongst scorers). Second, three scorers independently examined each participant to gauge whether the memories provided in their response were valid (i.e., actual memories, not keyboard smashes) and whether the response had similar keywords to the memories in another response. If two responses shared similar keywords across one or several memories, then the scorer compared the demographic information of the responses and made an overall judgment about whether the responses were duplicates. Responses were eliminated if at least two out of three scorers identified them as a duplicate. If a duplicate response was detected, we removed the second response (i.e., the response that was recorded at a later date).

We conducted a series of final checks to ensure the accuracy of our results. First, two researchers independently cleaned the data in R (each writing their own code), and the Qualtrics response identifiers in the data frames that each researcher generated were compared to ensure that both final data frames were identical (note that Qualtrics response identifiers are unique to each response, and are different from the subject identifiers we used to filter for duplicates). Second, two researchers independently wrote the code to analyze the data to ensure the numbers written in the manuscript were all correct.

### Narrative Hypotheses

In our preregistration of Study 1, we formulated two narrative hypotheses based on Levine et al.’s Autobiographical Interview scoring procedure (2002) that were conceptually related (but not identical) to our self-report hypotheses. To score our narratives, we fed our narratives through Van Genugten and Schacter’s (2024) automated scorer of the Autobiographical Interview. The automatic scoring tool uses a natural language model that was trained with hand-scored Autobiographical Interview data from several studies. Notably, the training data did not include data from repeated event memories, which is why we eventually determined that this tool was not the best fit for our data and may have led to inaccurate results.

Van Genugten & Schacter (2024) previously showed moderate to strong correlations between the automatic scorer and hand-scoring: *r* = .67 to .89 for episodic/internal content, and *r* = .33 to .80 for semantic/external content (Van Genugten & Schacter, 2024). Despite this, there are several key differences between the two procedures. First, the automated scorer analyzes narratives in terms of the predicted number of internal (i.e., episodic) versus external (i.e., non-episodic) words, instead of internal/external details (where a detail would contain several words). Second, the automated scorer is unable to distinguish between an internal event (i.e., the particular episode being scored) and an external event (i.e., an episode that is different from the episode being scored) meaning that both will be scored as internal content (Van Genugten & Schacter, 2024). Thus, while conceptually related, internal content encompasses several episodes and our measure of self-reported single episode reliance encompasses one episode, so these measures ought to be interpreted differently.

To ensure the narratives were processed correctly by the autoscorer, Oliver R. Bontkes (O.R.B.; who was previously trained on Autobiographical Interview scoring procedures) preprocessed all the narratives by fixing typos, adding punctuation, and removing unmatched quotes. Furthermore, to ensure that the output for external content corresponded more closely with semantic information, we removed all statements in the narratives that would fall under Levine et al.’s (2002) Autobiographical Interview scoring categories of “Repetition” (i.e., stating the same detail twice within a narrative) or “Other” (e.g., metacognitive statements, such as “I would still consider this activity to be a repeated event” or "thinking about the memory of doing laundry made me laugh”)); the removal of such content was preregistered. In total, we eliminated 16 statements from 15 narratives in Study 1, which was the only sample of narratives that we analyzed in this study (see Supplementary Results). After running the narratives through the automatic scorer, we calculated the proportion of internal and external content for each repeated event memory. Importantly, because internal and external content are mutually exclusive and exhaustive in the scoring, analyses using the proportion of external content directly mirror analyses using the proportion of internal content (i.e., we should have preregistered only one narrative hypothesis to prevent redundancy).

## Content Analysis of Memory Narratives

To help describe the diversity amongst repeated event memories in our sample, we investigated what kind of repeated event memories participants generated by coding narratives according to a) the central topic category, and b) whether the narrative was social or individual (note that this particular analytic plan was not preregistered). To generate a list of topic categories, a team of four research assistants independently coded one quarter of the narratives from Study 1 (i.e., 72-73 narratives). Their task was to generate topic categories that would fit the content of the narratives. O.R.B. then extracted categories that were common amongst coders and created broader thematic categories to capture any topics that were less common across coders. The resulting categories were: chores, exercise, recreation, school, transport, work, and a miscellaneous category for any topics that did not fit well into the other categories. To standardize the coding of narratives, O.R.B. created coding guidelines for the next phase of coding (see Guidelines for Content Analysis Coding).

In the next phase, the four research assistants and O.R.B. all coded 20% of the sample in Study 1 (i.e., 59 randomly selected narratives) to enable the calculation of inter-rater reliability estimates. The rest of the Study 1 sample was randomly divided amongst coders (i.e., 46-47 narratives per coder). To assess inter-rater reliability, we calculated Fleiss’s Kappa (Fleiss, 1971) using the irr package in R (Gamer et al., 2019). For Study 1, κ = .75 for both topic and social coding. Because coding of Study 1 narratives indicated substantial agreement, we proceeded with coding for Study 2, where we again randomly selected 20% of the narratives (i.e., 252 narratives) for reliability analyses and divided the rest of the narratives evenly amongst scorers (i.e., 201 narratives per scorer). For Study 2, κ = .79 for topic coding, and κ = .66 for social coding. Because social coding indicated lower reliability than in Study 1, we conducted pairwise reliability analyses to determine the source of increased variability across the coders. The results indicated lower reliability estimates for one coder: Cohen’s κ ranged from .43-49, while the lowest reliability amongst the other coders was .74. Inspection of their coding indicated that the coder inconsistently applied the guidance for social coding. Therefore, O.R.B. re-coded all their narratives from both Study 1 and Study 2, leading to an updated reliability estimate of κ = .80 for social coding in both studies. We elected to keep the original coder’s topic coding as they demonstrated strong reliability with other coders. Disagreements in the reliability sample were resolved by selecting the category that was most frequently selected amongst coders. We found ties in the coding of two memories in each study (e.g., two coders picked one category, two coders picked another category, and one coder picked yet another category). In the four cases this occurred, the function we used automatically selected coding of the first listed coder.

## *Guidelines for Content Analysis Coding*

Scoring guidelines for topic and social coding are reported verbatim as follows:

“There are 2 things you will be coding for: a) whether the event is social or individual (write “social” or “individual” in the “Social” column; b) the category of the event.

Regarding a) the decision about whether an event is social or not should be based on whether the narrator describes *interacting* with other people (i.e., it is **not** sufficient for other people to merely be present at the event; the person should be engaging in an actual social interaction for the event to be considered social). A social interaction in this case could be something like a conversation (could be as simple as ordering food) or doing an activity together (e.g., with a friend).

- Some examples of social repeated events would be: team sports, running/working out with friends, going to movies with other people, attending classes/lectures.
- Some examples of individual repeated events would be: chores, running or walking alone, playing video games (when alone)
- Some examples of events that would be considered individual despite other people being present could be: studying in a library or going to the gym

Regarding b) there are 7 possible categories:

1. Chores: events where the primary focus is some sort of task that is concerned with maintaining daily living standards, cleanliness, and/or organisation. These events are often but not always done out of a sense of duty or obligation. This category includes things like running errands. Some examples would be: cleaning, cooking, or grocery shopping.
2. Exercise: events where the primary focus is on some form of physical activity, usually done for health benefits. Some examples would be: going to the gym, running, swimming, or walking.
3. Recreation: events done for a sense of fun, entertainment, or relaxation when one is not working. Some examples would be: hobbies (such as dancing, playing music, or arts and crafts), sports, video games, or watching tv/movies.
4. School: events where the primary focus is on some sort of education-related activity. Some examples would be: attending lectures, studying/doing homework, or attending a study group.
5. Transport: events where the primary focus is on travelling from one place to another. Some examples would be: driving to school/work or taking the bus to school/work.
6. Work: events where the primary focus is on some sort of employment or volunteer-related activity, but not activities pertaining to one’s education. Some examples would be: attending meetings at work, volunteering for the food bank, or volunteering/working for a lab.
7. Miscellaneous: events that do not fit well into any of the above categories.

Some repeated event memories might fit into several categories, and in this case you should place it into the category that you believe best describes the event. Some potential ambiguities may be the following:

1. Exercise vs recreation when it comes to sports or physical activity done for recreational purposes. In general, the deciding factor should be whether the physical activity is primarily done in the pursuit of fun (e.g., with most sports, or things like hiking in nature) or if it is done primarily for health-related purposes (e.g., going to the gym or running).
2. Chores vs recreation when one finds enjoyment in doing their chores (e.g., if they enjoy cleaning or cooking or perhaps consider chores to be a form of self-care). The central consideration here should be whether the activity is important for maintaining daily living standards. A secondary consideration is whether the activity is whether the person goes above and beyond what would be considered normal duties in completing the chore. For example, cleaning will usually be a chore unless the person cleans things that don’t necessarily need to be cleaned just for the sake of cleaning. Cooking will be a more variable case: many people cook out of a sense of obligation (in which case it would be a chore) but many other people use cooking to be creative or to experiment with new recipes/flavours, in which case it could be considered recreational.
3. Exercise vs transport when it comes to something like biking to work (where one is exercising while travelling). These events would generally be considered exercise, particularly if an alternative that is faster and doesn’t involve physical activity exists (such as taking public transportation or driving instead of biking).”

## Supplementary Results

Figure S1 displays emotional valence histograms in Study 1 and Study 2, which we examined to gauge whether adding a note to the consent form in the Aberdeen sample asking not to recall stressful events affected the emotional valence distribution in Study 2 (see main text). Table S1 depicts three checks we conducted to ensure our results remained significant even when removing influential values (memories greater than three times the mean Cook’s Distance value), negative memories (memories with an emotional valence less than negative one), and old memories (memories where the last instance of the repeated event was not in the same year as the study–2022 for Study 1 and 2023 for Study 2).

**Figure S1**

*Emotional Valence Histograms for Study 1 and Study 2*


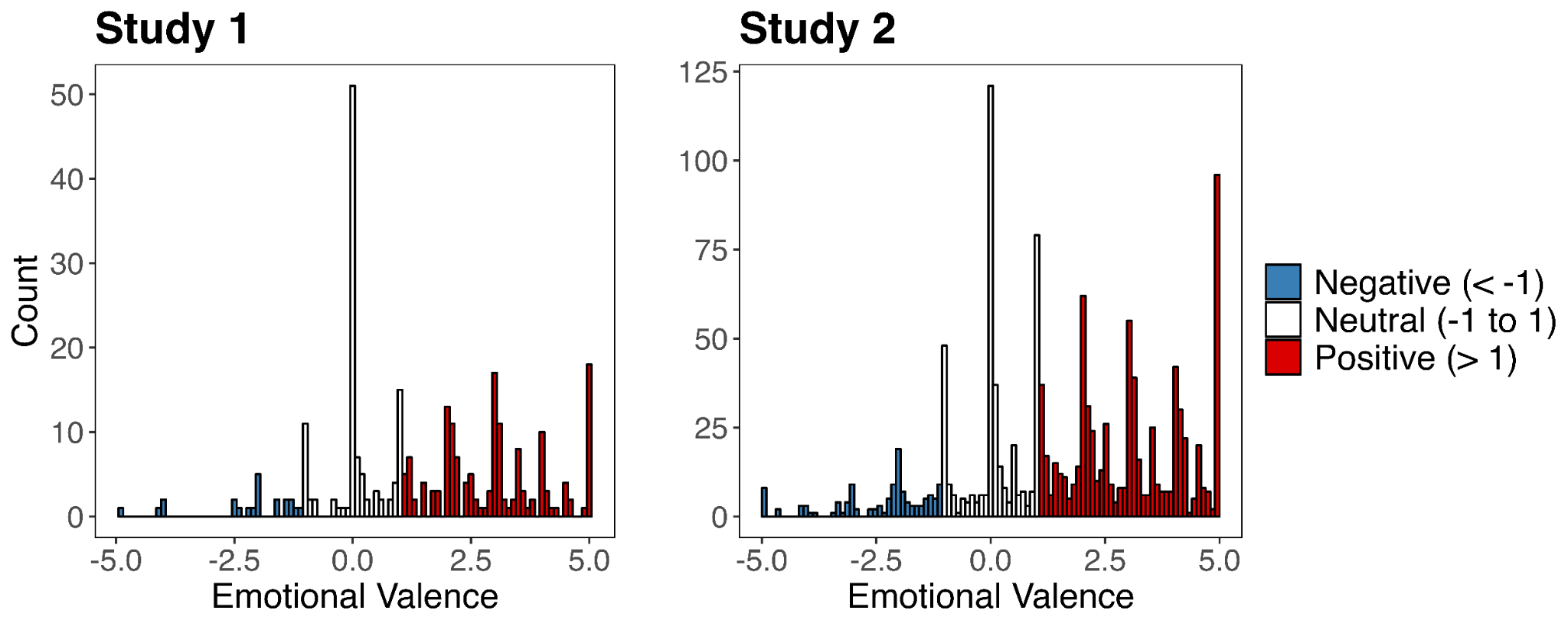


*Note*. We compared the emotional valence distributions in Study 1 and Study 2 to see whether adding a note in the Aberdeen consent form to not recall stressful events would impact the distribution of memories according to emotional valence.

**Table S1**

*Repeated Measures Correlations for Main Hypotheses with Exclusions of: Outliers (Cook's Distance, preregistered), Negative Memories, and Old Memories*

| Correlation | Study 1 | | | | Study 2 | | | |
| --- | --- | --- | --- | --- | --- | --- | --- | --- |
|  | *r_rm_* | *df* | 95% CI | *p* | *r_rm_* | *df* | 95% CI | *p* |
|  | Outliers Excluded | | | | | | | |
| Sim, Sem | .30 | 168 | [.16, .44] | < .001 | .35 | 720 | [.27, .41] | <.001 |
| Sim, SingEp | -.11 | 167 | [-.24, .02] | .137 | -.18 | 722 | [-.25, -.10] | <.001 |
|  | Negative Memories Excluded | | | | | | | |
| Sim, Sem | .24 | 171 | [.12, .37] | .002 | .24 | 715 | [.17, .31] | <.001 |
| Sim, SingEp | -.11 | 171 | [-.26, .04] | .145 | -.15 | 715 | [-.22, -.08] | <.001 |
|  | Old Memories Excluded | | | | | | | |
| Sim, Sem | .21 | 182 | [.07, .33] | .004 | .23 | 742 | [.16, .31] | <.001 |
| Sim, SingEp | -.12 | 182 | [-.27, .03] | .107 | -.11 | 742 | [-.19, -.05] | .002 |

*Note*. Sim = Overall Similarity. Sem = semantic reliance. SingEp = single episode reliance.

## Correlations Between Memory Reliance Variables and Similarity

In Figure S2, we present a correlation matrix between the three memory reliance variables (semantic, single episode, and mixed episodes) and the three similarity variables (overall similarity, similarity of place, and similarity of people). We were particularly interested in examining correlations between memory reliance variables and the similarity of place and similarity of people variables to gauge whether the more specific similarity measures are significantly related to semantic/single episode reliance (not preregistered). We also explored whether mixed episodes reliance was associated with similarity in any way, with no specific hypotheses (not preregistered). We used Holm’s (1979) method to correct p-values we obtained in these seven analyses. We did not examine the significance of correlations amongst semantic, single episode, and mixed episodes reliance or amongst overall similarity, similarity of place, and similarity of people, but we present the coefficients for these correlations in Figure S2 for completeness.

**Figure S2**

*Repeated Measures Correlation Heatmaps Between Memory Reliance Variables and Similarity Variables for Study 1 and Study 2*


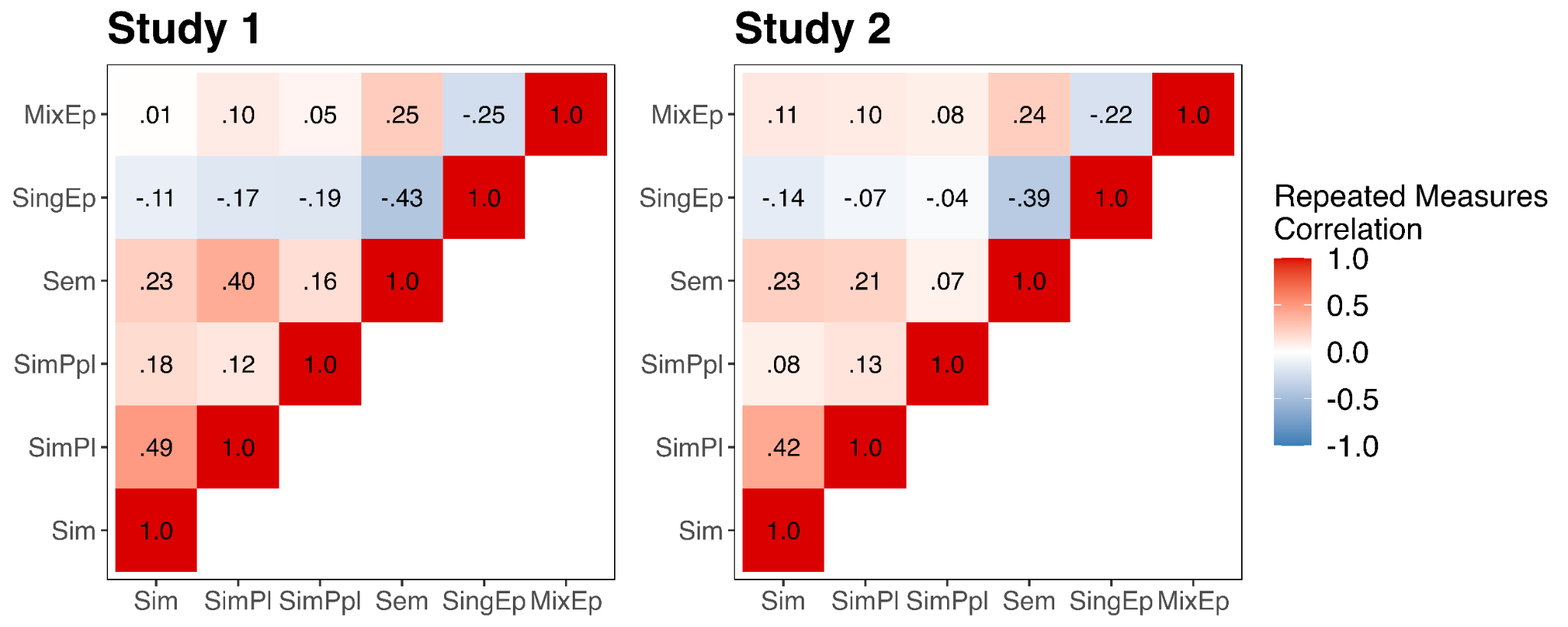


*Note*. MixEp = mixed episodes reliance. SingEp = single episode reliance. Sem = semantic reliance. SimPpl = similarity of people. SimPl = similarity of place. Sim = overall similarity. In Study 1, the only significant correlation after Holm’s correction was between semantic reliance and similarity of place.

From the correlations depicted in the left panel of Figure S2 (Study 1), two relationships in particular were of interest: a positive correlation between similarity of place and semantic reliance, *r_rm_*(193) = .40, *p_Holm_* < .001; and a negative correlation between similarity of place and episodic reliance, *r_rm_*(193) = -.17, *p_Holm_* = .087. These correlations were both larger than the corresponding correlations with overall similarity, although the correlation between similarity of place and episodic reliance was non-significant after applying Holm’s correction. We believed these findings, if replicated, would be important as they would suggest that certain subcomponents of similarity could be driving the association between overall similarity and semantic/episodic memory more than others.

In the Study 2 preregistration, we predicted that this pattern of results would replicate: Namely, that there would be a positive correlation between similarity of place and semantic reliance and a negative correlation between similarity of place and episodic reliance with both effect sizes larger than for memory reliance correlations with overall similarity. Both similarity of place correlations were significant in Study 2: for semantic reliance, *r_rm_*(837) = .21, [.15, 0.28], *p* < .001; and for episodic reliance, *r_rm_*(837) = -.07, [-.14, .00], *p* = .039. However, the effect sizes were not larger than those for the overall similarity measure (see Figure S2).

## Profile Analysis

### Study 1

Initially, we selected four profile models to examine further based on Bayesian Information Criterion (BIC) values (see Figure S3): Each model we examined was the highest performing model for a particular number of profiles. These profile models are differentiated based on their distribution structure and whether the volume, shape, and/or orientation of the covariances are held equal or are variable across profiles (see Scrucca et al., 2016). The four models we selected to evaluate further were a two profile model with an ellipsoidal distribution structure, varying volume, varying shape, and varying orientation (VVV), a three profile model with an ellipsoidal distribution structure varying volume, equal shape, and varying orientation (VEV), a four profile model with a diagonal distribution structure, varying volume, and equal shape, with the orientation set on coordinate axes (VEI), and a five profile model with a spherical distribution structure (and therefore no orientation parameter), variable volume, and equal shape (VII). We did not examine a six profile model as this is where successive increases in BIC values taper off, indicating that there are no substantial gains in model fit being made when penalizing for decreased parsimony.

To evaluate these models further, we first looked at the integrated complete-data likelihood criterion (ICL; see Figure S4). The two profile, three profile, and four profile models were the highest performing models for their respective number of profiles. The five profile model indicated a relatively high cluster overlap when compared to other five profile models.

The next step in model evaluation was to directly compare the gains in model fit amongst neighboring profiles using bootstrapped likelihood ratio tests (BLRT). Unfortunately, we could only conduct BLRTs on the two profile and three profile models, as the function we used would not finish running for the four profile or five profile models even when reducing the number of bootstrap replications from 999 to 100 (indicating that these models are not stable/interpretable). For the two profile model, the BLRT indicated significant gains when moving from one profile to two profiles and when moving from two profiles to three profiles. For the three profile model, the BLRT indicated significant gains when moving from one profile to two profiles and when moving from two profiles to three profiles; less substantial but still significant gains were made when moving from three profiles to four profiles.

When examining the shape of the profiles in the different models, the two profile model was the only model that had profiles with noticeably distinct shapes: Profile one was balanced on the three memory reliance variables, and profile two was high on semantic and mixed episodes reliance but low on single episode reliance (we called this profile “low episodic reliance”). When moving to the three profile model, the balanced profile was split into two profiles: one with overall lower and one with overall higher ratings on all three memory reliance variables (which we termed “low overall reliance” and “high overall reliance,” respectively). The four profile model had essentially the same profiles as the three profile model, but added a small profile (*n* = 5 memories) that appeared to be a more extreme version of the low episodic reliance profile: It was very high on semantic and mixed episodes reliance and very low on single episode reliance. The five profile model added a further split of the balanced profiles, and also had the small profile from the four profile model. See Table S2 for model fit indices of selected models and their neighbors, and see Table S3 for sample sizes, means, and standard deviations of all the profiles in the four models we analyzed. Figures S5, S6, and S7 are graphical depictions of the two, four, and five profile models (see Figure 3 in the main text for a graphical depiction of the three profile model). To inspect models other than those examined here, open data and code for Study 1 and Study 2 are available at [osf.io/hxrc6](http://osf.io/hxrc6).

**Figure S3**

*Study 1 BIC Values for 14 mclust Model Types From One to Nine Profiles*

*
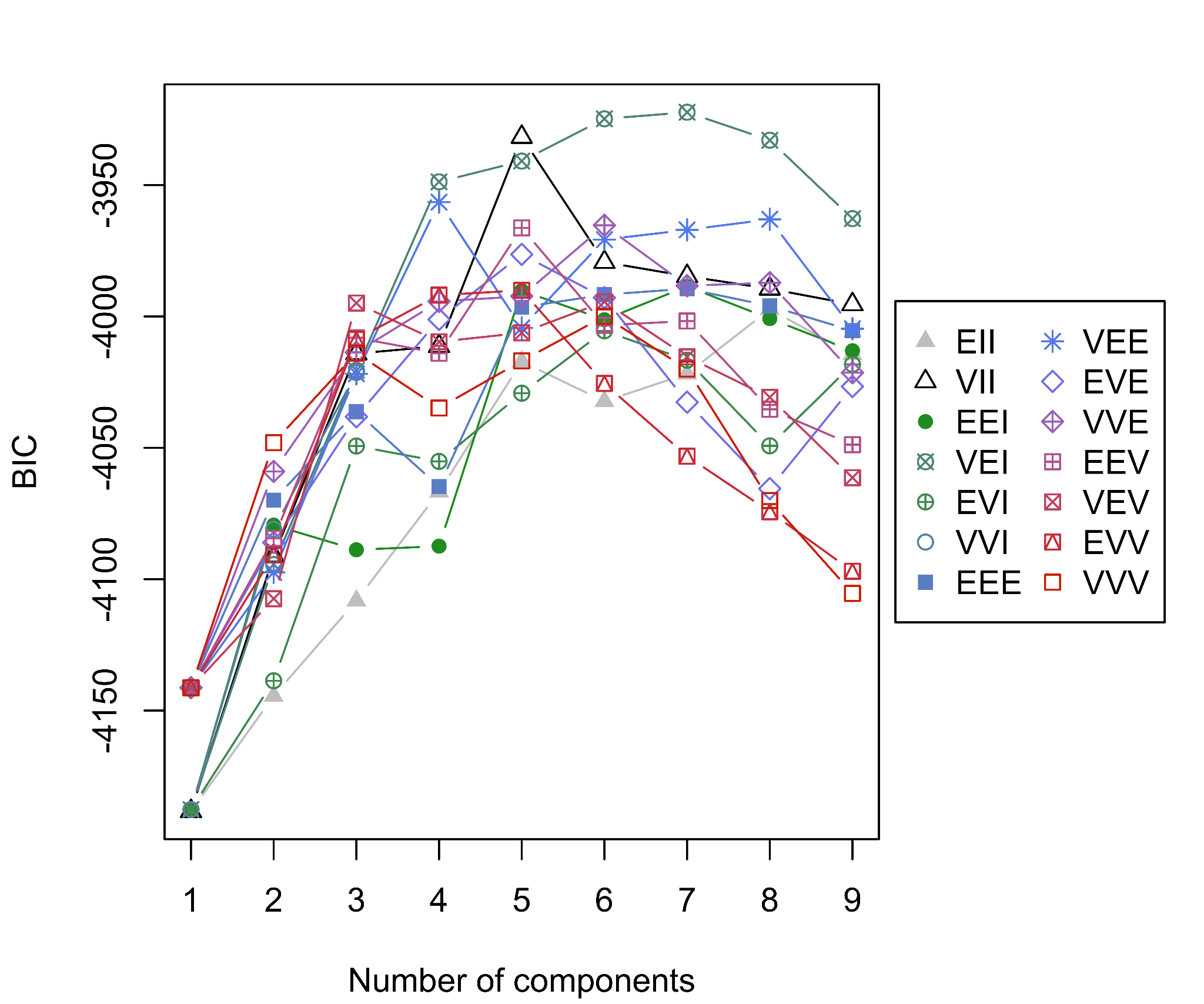
*

*Note.* Typically the goal is to minimize the BIC value when fitting a model, but in the mclust package the BIC value is a negative version of the normal BIC so in this case the goal is to maximize the BIC value.

**Figure S4**

*Study 1 ICL Values for 14 mclust Model Types From One to Nine Profiles*


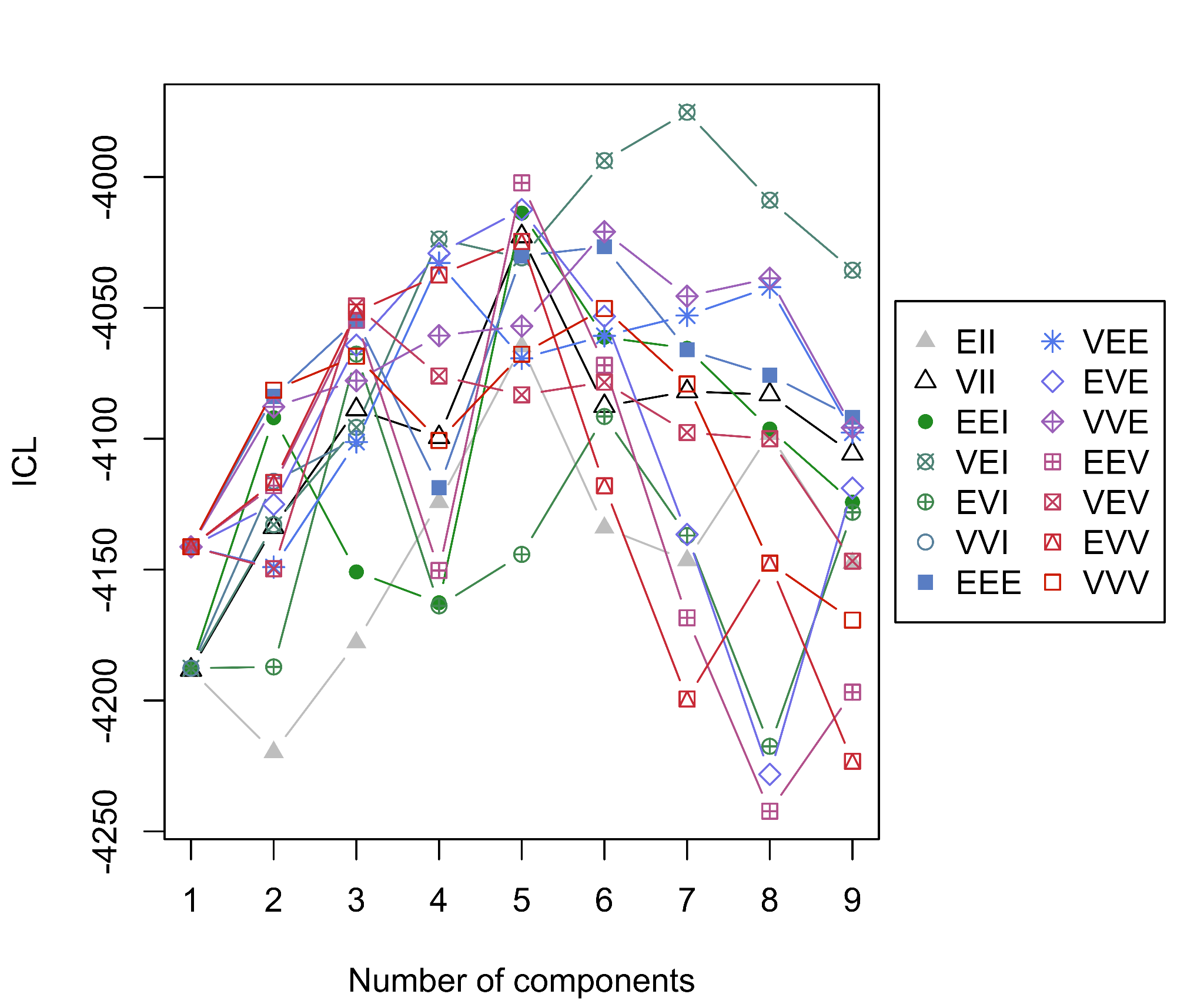


**Table S2**

*Study 1 Model Fit Indices for Selected Models and Neighboring Models*

| Number of Profiles | BIC | ICL | BLRT Statistic | BLRT *p* Value | BLRT Meaning | Smallest *n* Proportion |
| --- | --- | --- | --- | --- | --- | --- |
| VVV Model Type | | | | | | |
| 1 | -4141.30 | -4141.30 | 150.00 | < .001 | 2 > 1 | 1.00 |
| 2 | -4048.04 | -4081.46 | 90.85 | < .001 | 3 > 2 | .35 |
| VEV Model Type | | | | | | |
| 1 | -4141.30 | -4141.30 | 79.26 | < .001 | 2 > 1 | 1.00 |
| 2 | -4107.42 | -4149.67 | 157.90 | < .001 | 3 > 2 | .31 |
| **3** | **-3994.91** | **-4049.26** | **30.65** | **.003** | 4 > 3 | **.25** |
| VEI Model Type | | | | | | |
| 1 | -4187.67 | -4187.67 | – | – | – | 1.00 |
| 2 | -4094.41 | -4132.84 | – | – | – | .30 |
| 3 | -4020.50 | -4095.68 | – | – | – | .31 |
| 4 | -3948.77 | -4023.69 | – | – | – | .02 |
| VII Model Type | | | | | | |
| 1 | -4188.30 | -4188.30 | – | – | – | 1.00 |
| 2 | -4091.10 | -4133.84 | – | – | – | .30 |
| 3 | -4014.12 | -4088.78 | – | – | – | .32 |
| 4 | -4011.32 | -4099.41 | – | – | – | .09 |
| 5 | -3931.71 | -4022.71 | – | – | – | .02 |

*Note*. The bolded row indicates the model that we selected. Normally the goal is to minimize BIC values but in the mclust package the BIC value is a negative version of the normal BIC, so in this case the goal is to maximize the BIC value (this applies to ICL values as well, which adds a penalty for entropy to BIC values). The BLRT compares the current model with a model with k+1 profiles, and significant *p* values indicate that the model with k profiles ought to be rejected in favor of the model with k+1 profiles. For the VEI and VII model types, BLRTs would not finish running, which is why there is no BLRT data for these two model types.

**Table S3**

*Study 1 Profiles in Four Different Models*

| Profile |  | Semantic Reliance | | Single Episode Reliance | | Mixed Episodes Reliance | |
| --- | --- | --- | --- | --- | --- | --- | --- |
|  | *n* | *M* | *SD* | *M* | *SD* | *M* | *SD* |
| Two Profile VVV Model | | | | | | | |
| 1 | 101 | 8.82 | 0.97 | 1.83 | 1.21 | 7.00 | 2.60 |
| 2 | 190 | 5.90 | 2.31 | 6.40 | 2.24 | 6.16 | 2.50 |
| Three Profile VEV Model | | | | | | | |
| 1 | 104 | 8.49 | 1.16 | 2.19 | 1.23 | 7.76 | 1.59 |
| 2 | 113 | 6.88 | 1.61 | 7.15 | 1.01 | 7.14 | 1.54 |
| 3 | 74 | 4.76 | 2.94 | 4.94 | 3.45 | 3.57 | 2.70 |
| Four Profile VEI Model | | | | | | | |
| 1 | 96 | 8.42 | 1.15 | 2.24 | 1.11 | 7.66 | 1.51 |
| 2 | 97 | 7.24 | 1.31 | 7.16 | 1.03 | 7.43 | 1.43 |
| 3 | 93 | 4.86 | 2.77 | 5.28 | 3.20 | 4.00 | 2.64 |
| 4 | 5 | 10.00 | 0.00 | 0.02 | 0.04 | 10.00 | 0.00 |
| Five Profile VII Model | | | | | | | |
| 1 | 97 | 8.40 | 1.14 | 2.28 | 1.16 | 7.72 | 1.48 |
| 2 | 75 | 6.22 | 1.14 | 7.03 | 1.06 | 6.51 | 1.16 |
| 3 | 82 | 4.98 | 3.00 | 5.02 | 3.32 | 3.79 | 2.73 |
| 4 | 5 | 10.00 | 0.00 | 0.02 | 0.04 | 10.00 | 0.00 |
| 5 | 32 | 8.49 | 0.89 | 7.52 | 0.87 | 8.74 | 0.96 |

*Note.* Profile numbers are intended to correspond between models (i.e., the same number represents roughly the same profile across models). Profile 1 roughly corresponds to the low episodic reliance profile in the main text, profile 2 corresponds with the high overall reliance profile, and profile 3 corresponds with the low overall reliance profile.

**Figure S5**

*Study 1 Mean Memory Reliance Ratings in the Two Profile Model*

*
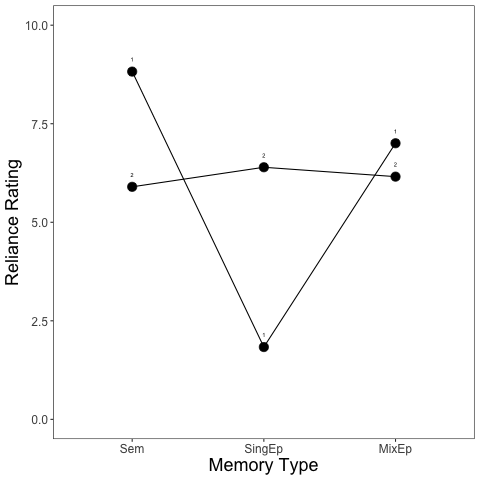
*

*Note*. Sem = semantic reliance. SingEp = single episode reliance. MixEp = mixed episodes reliance.

**Figure S6**

*Study 1 Mean Memory Reliance Ratings in the Four Profile Model*


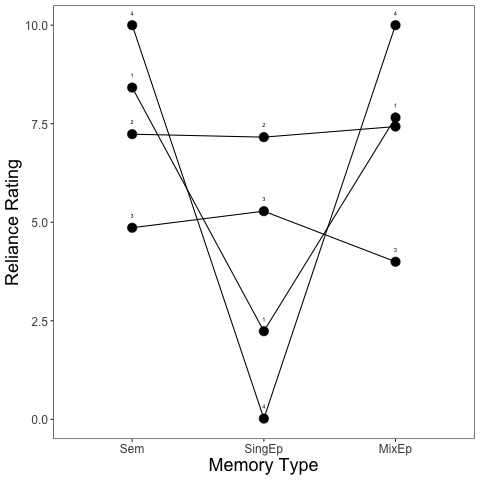


*Note*. Sem = semantic reliance. SingEp = single episode reliance. MixEp = mixed episodes reliance.

**Figure S7**

*Study 1 Mean Memory Reliance Ratings in the Five Profile Model*

*
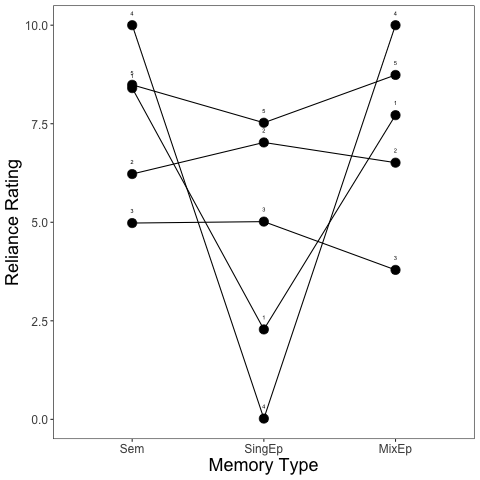
*

*Note*. Sem = semantic reliance. SingEp = single episode reliance. MixEp = mixed episodes reliance.

###

### Study 2

We performed an exploratory profile analysis in Study 2 with the same procedure as in Study 1. We first selected three of the highest performing models based on BIC values depicted in Figure S8: a two profile model with the same VVV structure as Study 1, a three profile model also with the same VEV structure as Study 1, and a five profile model with an ellipsoidal distribution structure, variable volume, variable shape, and equal orientation (VVE). We did not examine a four profile model as the highest performing four profile model had a lower BIC value than the highest performing three profile model. We did not conduct further analyses on any six profile models as this is where successive increases in BIC values taper off. When evaluating ICL values (Figure S9), both the two profile and three profile models were still the highest performing models for their respective number of profiles. However, the five profile model performed poorer than one other five profile model, indicating that there was relatively higher cluster overlap in this model.

For the two profile model, the BLRT indicated significant gains when moving from one profile to two profiles and when moving from two profiles to three profiles. For the three profile model, the BLRT indicated significant gains when moving from one profile to two profiles and when moving from two profiles to three profiles; less substantial but still significant gains were made when moving from three profiles to four profiles. For the five profile model, the BLRT indicated significant gains when moving from one to two profiles, two to three profiles, three to four profiles, and four to five profiles but there were not significant gains when moving from five profiles to six profiles.

The two profile model had one profile that was balanced across the three memory reliance variables and one profile that was high on semantic and mixed episodes reliance but medium on single episode reliance. The three profile model was very similar to the three profile model in Study 1. The five profile model had mainly similar profiles to those in the three profile model: It had three profiles with relatively balanced means on all three memory reliance variables and one profile which was high on semantic and mixed episodes reliance and low on single episode reliance. However, the fifth profile in this model had a novel shape that we did not observe in any of the other models which had very high mean ratings on semantic reliance and very low mean ratings on single episode and mixed episode reliance. Table S4 displays model fit indices for selected models and their neighbors. Table S5 depicts the sample sizes, means, and standard deviations of all the profiles in the three models we analyzed. See Figure S10 and S11 for graphical depictions of the model two and five profile models, and see Figure 3 in the main text for a depiction of the three profile model.

**Figure S8**

*Study 2 BIC Values for the 14 mclust Model Types From One to Nine Profiles*


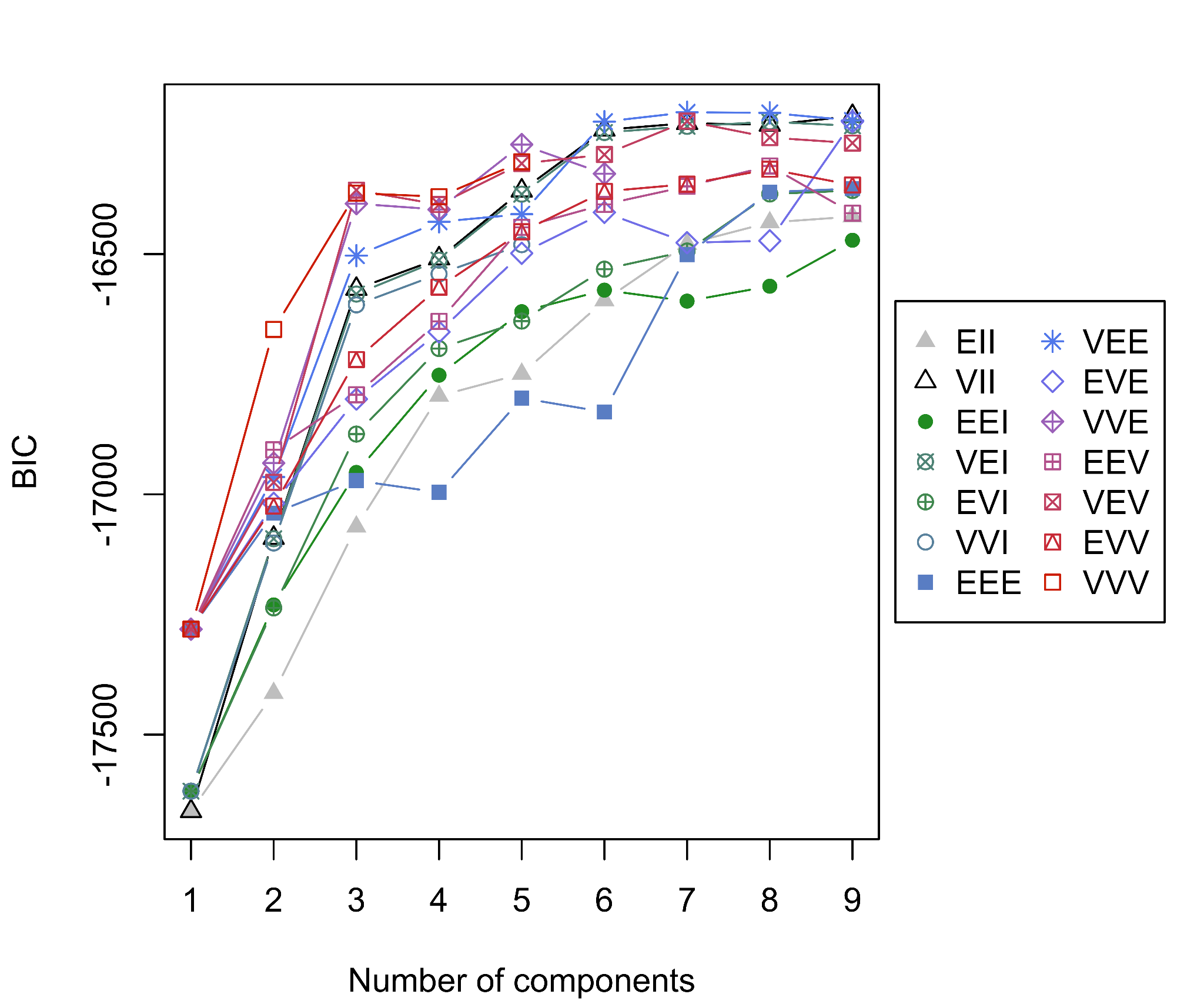


*Note.* Usually the goal is to minimize the BIC value when fitting a model, but in the mclust package the BIC value is a negative version of the normal BIC so in this case the goal is to maximize the BIC value.

**Figure S9**

*Study 2 ICL Values for the 14 mclust Model Types From One to Nine Profiles*


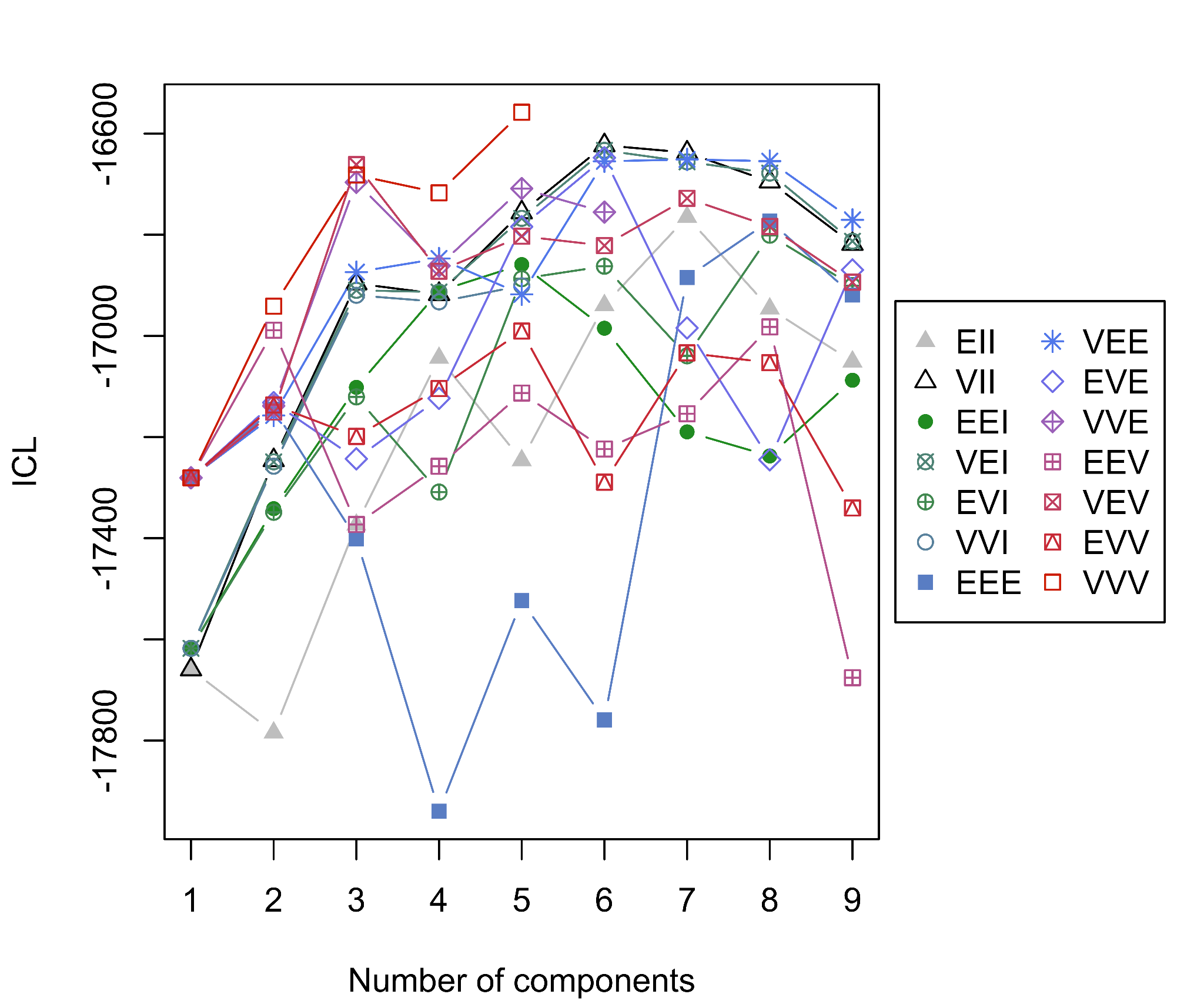


**Table S4**

*Study 2 Model Fit Indices for Selected Models and Neighboring Models*

| Number of Profiles | BIC | ICL | BLRT Statistic | BLRT *p* Value | BLRT Meaning | Smallest *n* Proportion |
| --- | --- | --- | --- | --- | --- | --- |
| VVV Model Type | | | | | | |
| 1 | -17280.57 | -17280.57 | 695.15 | < .001 | 2 > 1 | 1.00 |
| 2 | -16656.79 | -16941.19 | 354.97 | < .001 | 3 > 2 | .32 |
| VEV Model Type | | | | | | |
| 1 | -17280.57 | -17280.57 | 362.69 | < .001 | 2 > 1 | 1.00 |
| 2 | -16974.96 | -17150.67 | 665.15 | < .001 | 3 > 2 | .26 |
| **3** | **-16366.91** | **-16661.29** | **27.11** | **.003** | **4 > 3** | **.31** |
| VVE Model Type | | | | | | |
| 1 | -17280.57 | -17280.57 | 395.68 | < .001 | 2 > 1 | 1.00 |
| 2 | -16934.84 | -17138.25 | 589.64 | < .001 | 3 > 2 | .23 |
| 3 | -16395.15 | -16696.21 | 37.80 | .002 | 4 > 3 | .31 |
| 4 | -16407.31 | -16861.23 | 185.13 | < .001 | 5 > 4 | .16 |
| 5 | -16272.13 | -16708.69 | -10.73 | .986 | 5 > 6 | .03 |

*Note*. The bolded row indicates the model that we selected. Normally the goal is to minimize BIC values but in the mclust package the BIC value is a negative version of the normal BIC, so in this case the goal is to maximize the BIC value (this applies to ICL values as well, which adds a penalty for entropy to BIC values). The BLRT compares the current model with a model with k+1 profiles, and significant *p* values indicate that the model with k profiles ought to be rejected in favor of the model with k+1 profiles.

**Table S5**

*Study 2 Profiles in Three Different Models*

| Profile |  | Semantic Reliance | | Single Episode Reliance | | Mixed Episodes Reliance | |
| --- | --- | --- | --- | --- | --- | --- | --- |
|  | *n* | *M* | *SD* | *M* | *SD* | *M* | *SD* |
| Two Profile VVV Model | | | | | | | |
| 1 | 849 | 7.83 | 1.28 | 4.84 | 2.65 | 7.88 | 1.23 |
| 2 | 408 | 5.31 | 2.84 | 5.36 | 3.02 | 4.39 | 2.68 |
| Three Profile VEV Model | | | | | | | |
| 1 | 400 | 8.39 | 1.14 | 2.28 | 1.26 | 8.20 | 1.23 |
| 2 | 473 | 7.29 | 1.27 | 6.97 | 1.11 | 7.55 | 1.22 |
| 3 | 384 | 5.23 | 2.85 | 5.43 | 3.09 | 4.25 | 2.65 |
| Five Profile VVI Model | | | | | | | |
| 1 | 396 | 8.48 | 1.05 | 2.31 | 1.30 | 8.15 | 1.24 |
| 2 | 224 | 6.72 | 0.67 | 6.69 | 0.74 | 6.97 | 0.76 |
| 3 | 402 | 4.87 | 2.44 | 5.93 | 2.72 | 4.91 | 2.61 |
| 4 | 195 | 8.30 | 1.04 | 7.49 | 1.12 | 8.49 | 1.00 |
| 5 | 40 | 9.39 | 0.65 | 1.00 | 1.00 | 1.58 | 1.17 |

*Note.* Profile numbers are intended to correspond between models (i.e., the same number represents roughly the same profile across models). Profile 1 roughly corresponds to the low episodic reliance profile in the main text, profile 2 corresponds with the high overall reliance profile, and profile 3 corresponds with the low overall reliance profile.

**Figure S10**

*Study 2 Mean Memory Reliance Ratings in the Two Profile Model*

*
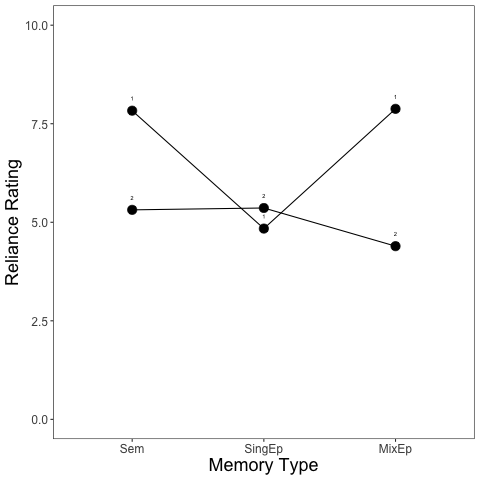
*

*Note*. Sem = semantic reliance. SingEp = single episode reliance. MixEp = mixed episodes reliance.

**Figure S11**

*Study 2 Mean Memory Reliance Ratings in the Five Profile Model*

*
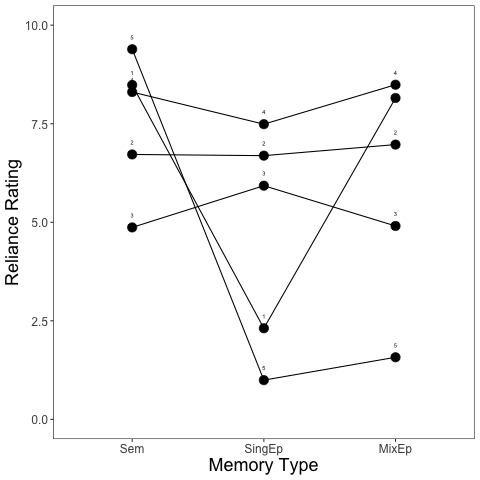
*

*Note*. Sem = semantic reliance. SingEp = single episode reliance. MixEp = mixed episodes reliance.

###

### Mixed-Effect Analyses

Table S6 lists regression statistics for the mixed-effect analyses we ran in Study 1 and Study 2, which examined whether there are significant differences between the profiles in similarity, similarity of place, similarity of people, vividness, and frequency. We report additional models where we added controls in the analyses of our similarity variables to determine whether one variable in particular might be driving results. The low episodic reliance profile was the main reference group in these analyses, but we also compared the high overall reliance and low overall reliance profiles, using the high overall reliance profile as the reference group.

**Table S6**

*Study 1 and Study 2 Regression Statistics for Mixed-Effect Models*

|  | Study 1 | | | | Study 2 | | | |
| --- | --- | --- | --- | --- | --- | --- | --- | --- |
| Comparison | *b* | CI | *t* | *p* | *b* | CI | *t* | *p* |
| Overall Similarity ~ Profile | | | | | | | | |
| Low Epi vs High | 0.12 | [-0.51, 0.74] | 0.36 | .718 | -0.36 | [-0.68, -0.05] | -2.24 | .025 |
| Low Epi vs Low | -0.64 | [-1.34, 0.06] | -1.79 | .075 | -0.52 | [-0.85, -0.19] | -3.06 | .002 |
| High vs Low | -0.76 | [-1.45, -0.06] | -2.13 | .034 | -0.16 | [-0.47, 0.16] | -0.97 | .332 |
| Overall Similarity ~ Similarity of Place + Profile | | | | | | | | |
| SimPl | 0.36 | [0.26, 0.45] | 7.60 | < .001 | 0.36 | [0.31, 0.41] | 14.71 | < .001 |
| Low Epi vs High | 0.41 | [ -0.17, 1.00] | 1.39 | .165 | -0.19 | [ -0.49, 0.10] | -1.28 | .202 |
| Low Epi vs Low | -0.14 | [-0.80, 0.52] | -0.41 | .681 | -0.26 | [-0.57, 0.05] | -1.66 | .097 |
| High vs Low | -0.55 | [-1.20, 0.09] | -1.67 | .095 | -0.07 | [-0.36, 0.22] | -0.47 | .635 |
| Overall Similarity ~ Similarity of People + Profile | | | | | | | | |
| SimPpl | 0.17 | [0.07, 0.26] | 3.32 | .001 | 0.08 | [0.04, 0.13] | 3.41 | < .001 |
| Low Epi vs High | 0.14 | [-0.52, 0.80] | 0.41 | .680 | -0.28 | [-0.61, 0.05] | -1.64 | .102 |
| Low Epi vs Low | -0.57 | [-1.33, 0.20] | -1.45 | .149 | -0.43 | [-0.79, -0.07] | -2.36 | .019 |
| High vs Low | -0.71 | [-1.46, 0.05] | -1.84 | .068 | -0.15 | [-0.49, 0.19] | -0.87 | .386 |
| Similarity of Place ~ Profile | | | | | | | | |
| Low Epi vs High | -0.89 | [-1.57, -0.20] | -2.53 | .012 | -0.53 | [-0.85, -0.20] | -3.18 | .002 |
| Low Epi vs Low | -1.37 | [-2.14, -0.60] | -3.49 | < .001 | -0.71 | [-1.05, -0.37] | -4.09 | < .001 |
| High vs Low | -0.48 | [-1.24, 0.28] | -1.25 | .213 | -0.19 | [-0.51, 0.14] | -1.12 | .264 |
| Similarity of Place ~ Overall Similarity + Profile | | | | | | | | |
| Sim | 0.44 | [0.31, 0.56] | 7.10 | < .001 | 0.40 | [0.34, 0.45] | 14.45 | < .001 |
| Low Epi vs High | -0.96 | [-1.60, -0.31] | -2.88 | .004 | -0.37 | [-0.68, -0.07] | -2.41 | .016 |
| Low Epi vs Low | -1.16 | [-1.89, -0.42] | -3.09 | .002 | -0.52 | [-0.84, -0.20] | -3.20 | .001 |
| High vs Low | -0.20 | [-0.92, 0.52] | -0.54 | .589 | -0.15 | [-0.45, 0.16] | -0.95 | .343 |
| Similarity of Place ~ Similarity of People + Profile | | | | | | | | |
| SimPpl | 0.07 | [-0.04, 0.19] | 1.28 | .204 | 0.14 | [0.09, 0.19] | 5.22 | < .001 |
| Low Epi vs High | -0.93 | [-1.68, -0.17] | -2.40 | .017 | -0.61 | [-0.95, -0.26] | -3.41 | < .001 |
| Low Epi vs Low | -1.38 | [-2.26, -0.51] | -3.08 | .002 | -0.64 | [-1.02, -0.27] | -3.37 | < .001 |
| High vs Low | -0.46 | [-1.31, 0.40] | -1.04 | .298 | -0.04 | [-0.40, 0.32] | -0.21 | .833 |
| Similarity of People ~ Profile | | | | | | | | |
| Low Epi vs High | -0.28 | [-1.13, 0.57] | -0.65 | .516 | -0.13 | [-0.54, 0.28] | 0.61 | .545 |
| Low Epi vs Low | -1.24 | [-2.21, -0.27] | -2.50 | .013 | -0.52 | [-0.96, -0.08] | -2.33 | .020 |
| High vs Low | -0.95 | [-1.91, 0.00] | -1.95 | .052 | -0.40 | [-0.82, 0.03] | -1.84 | .066 |
| Similarity of People ~ Overall Similarity + Profile | | | | | | | | |
| Sim | 0.27 | [0.11, 0.42] | 3.39 | < .001 | 0.13 | [0.06, 0.21] | 3.53 | < .001 |
| Low Epi vs High | -0.30 | [-1.13, 0.53] | -0.71 | .477 | -0.09 | [-0.50, 0.32] | -0.43 | .670 |
| Low Epi vs Low | -1.04 | [-1.99, -0.09] | -2.13 | .034 | -0.47 | [-0.90, -0.03] | -2.08 | .038 |
| High vs Low | -0.74 | [-1.68, 0.20] | -1.53 | .127 | -0.38 | [-0.79, 0.04] | -1.76 | .078 |
| Similarity of People ~ Similarity of Place + Profile | | | | | | | | |
| SimPl | .09 | [-0.05, 0.23] | 1.27 | .206 | 0.18 | [0.11, 0.25] | 5.13 | < .001 |
| Low Epi vs High | -0.20 | [-1.06, 0.66] | -0.46 | .649 | -0.02 | [-0.43, 0.39] | -0.10 | .922 |
| Low Epi vs Low | -1.10 | [-2.09, -0.12] | -2.18 | .030 | -0.39 | [-0.83, 0.05] | -1.76 | .080 |
| Arousal ~ Profile | | | | | | | | |
| Low Epi vs High | 0.94 | [0.26, 1.62] | 2.74 | .007 | 0.56 | [0.20, 0.91] | 3.06 | .002 |
| Low Epi vs Low | -0.18 | [-0.94, 0.58] | -0.47 | .638 | -0.05 | [-0.42, 0.33] | -0.25 | .803 |
| High vs Low | -1.12 | [-1.90, -0.36] | -2.95 | .003 | -0.60 | [-0.96, -0.24] | -3.32 | < .001 |
| Frequency ~ Profile | | | | | | | | |
| Low Epi vs High | -0.09 | [-1.03, 0.86] | -0.18 | .858 | -0.13 | [-0.64, 0.38] | -0.51 | .610 |
| Low Epi vs Low | 0.81 | [-0.25, 1.87] | 1.50 | .135 | -0.18 | [-0.71, 0.36] | -0.65 | .517 |
| High vs Low | 0.90 | [-0.16, 1.96] | 1.66 | .099 | -0.04 | [-0.55, 0.46] | -0.17 | .865 |
| Vividness ~ Profile | | | | | | | | |
| Low Epi vs High | 0.09 | [-0.43, 0.61] | 0.34 | .732 | 0.17 | [-0.10, 0.43] | 1.22 | .222 |
| Low Epi vs Low | -0.64 | [-1.25, -0.04] | -2.13 | .034 | -0.03 | [-0.31, 0.24] | -0.24 | .808 |
| High vs Low | -0.73 | [-1.33, -0.13] | -2.44 | .016 | -0.20 | [-0.46, 0.06] | -1.48 | .138 |

*Note.* We used the following abbreviations in this table: Low Epi = low episodic reliance profile, High = high overall reliance profile, Low = low overall reliance profile, Sim = overall similarity, SimPl = similarity of place, and SimPpl = similarity of people.

###

### Cumulative Profile Analysis

Due to the fact that neither of our samples reached Nylund et al.’s (2007) recommendation for 500 subjects in order to be confident in the resulting profiles, we combined our data post-hoc and reran profiles analyses on the full sample of 1548 memories (516 subjects). BIC analysis that we ran on the full dataset indicated that the best two profile model type had a VVE structure, the best three profile model had a VEV structure, and the best four profile model had a VVE structure, with BIC values tapering off in models with five or more profiles (see Figure S12). The three profile models both had the highest ICL values compared to other models with the same number of profiles while the two profile VVE model had lower ICL values than four other two profile models and the four profile VVE model had lower ICL values than two other four profile models, indicating there was relatively high cluster overlap in these models compared to other model types with the same number of profiles (see Figure S13).

For the two profile model, the BLRT indicated significant gains when moving from one profile to two profiles and when moving from two profiles to three profiles. For the three profile model, the BLRT indicated significant gains when moving from one profile to two profiles, two profiles to three profiles, and when moving from three profiles to four profiles. For the four profile model, the BLRT indicated significant gains when moving from one to two profiles, two to three profiles, three to four profiles, and four to five profiles.

When evaluating the shape of the profiles, the two profile model had one profile that was high on semantic and mixed episodes reliance but low on single episode reliance and one balanced profile, similar to the two profile model in Study 1. The three profile model was very similar to the three profile models in Study 1 and Study 2. The four profile model was similar to the three profile model, but had one small profile (<5% of the full sample) that was high on semantic reliance and low on single episode and mixed episodes reliance. Table S7 displays model fit indices for the selected profiles and their neighbors, and Table S8 depicts the sample sizes, means, and standard deviations of all the profiles in the three models we analyzed. See Figure S14, S15, and S16 for graphical depictions of the two, three, and four profile models respectively. We determined the three profile model to be the best model as it had better model fit than the two profile model and it had no small profiles, unlike the four profile model.

**Figure S12**

*Cumulative BIC Values for the 14 mclust Model Types From One to Nine Profiles*

*
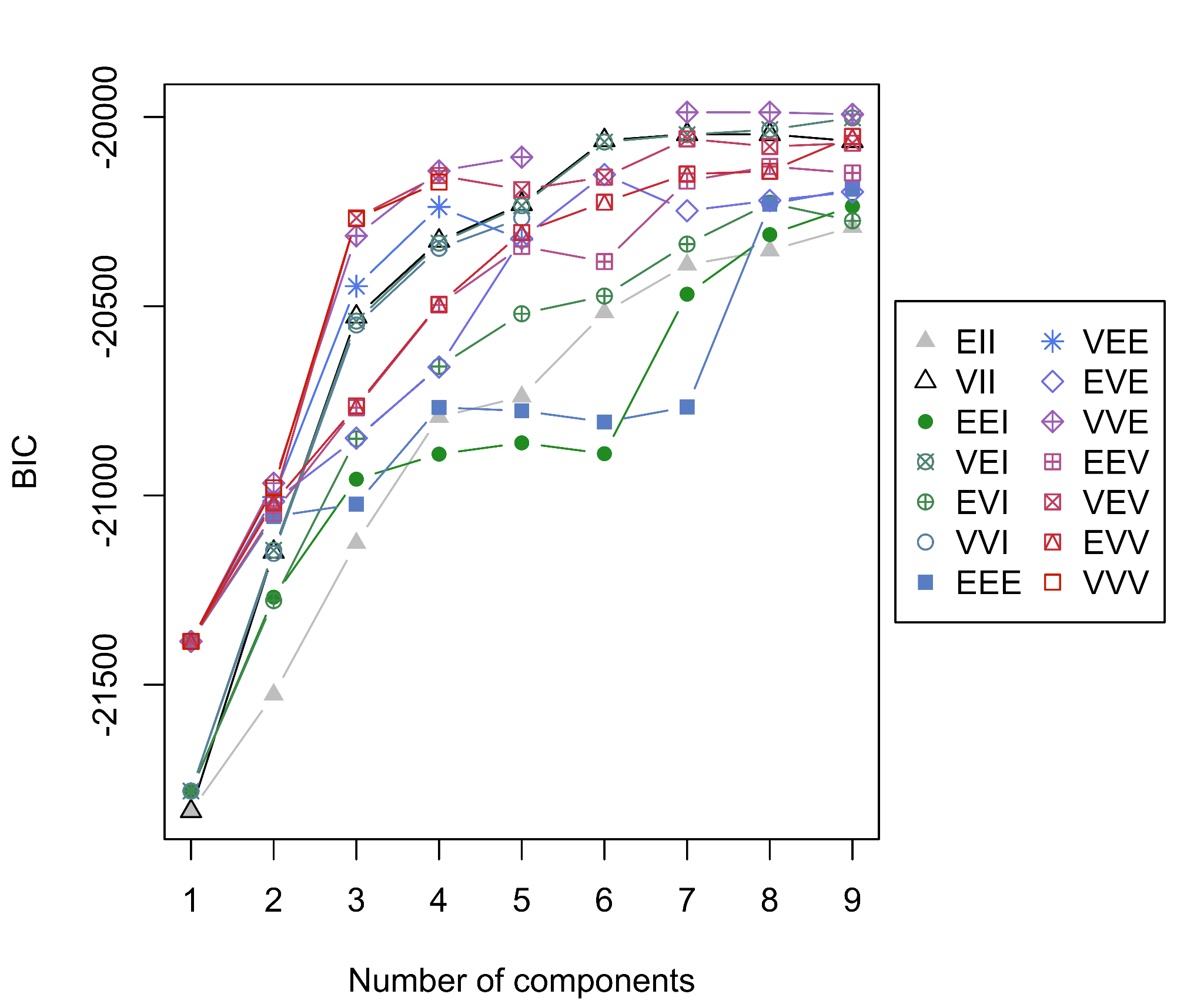
*

**Figure S13**

*Cumulative ICL Values for the 14 mclust Model Types From One to Nine Profiles*

**
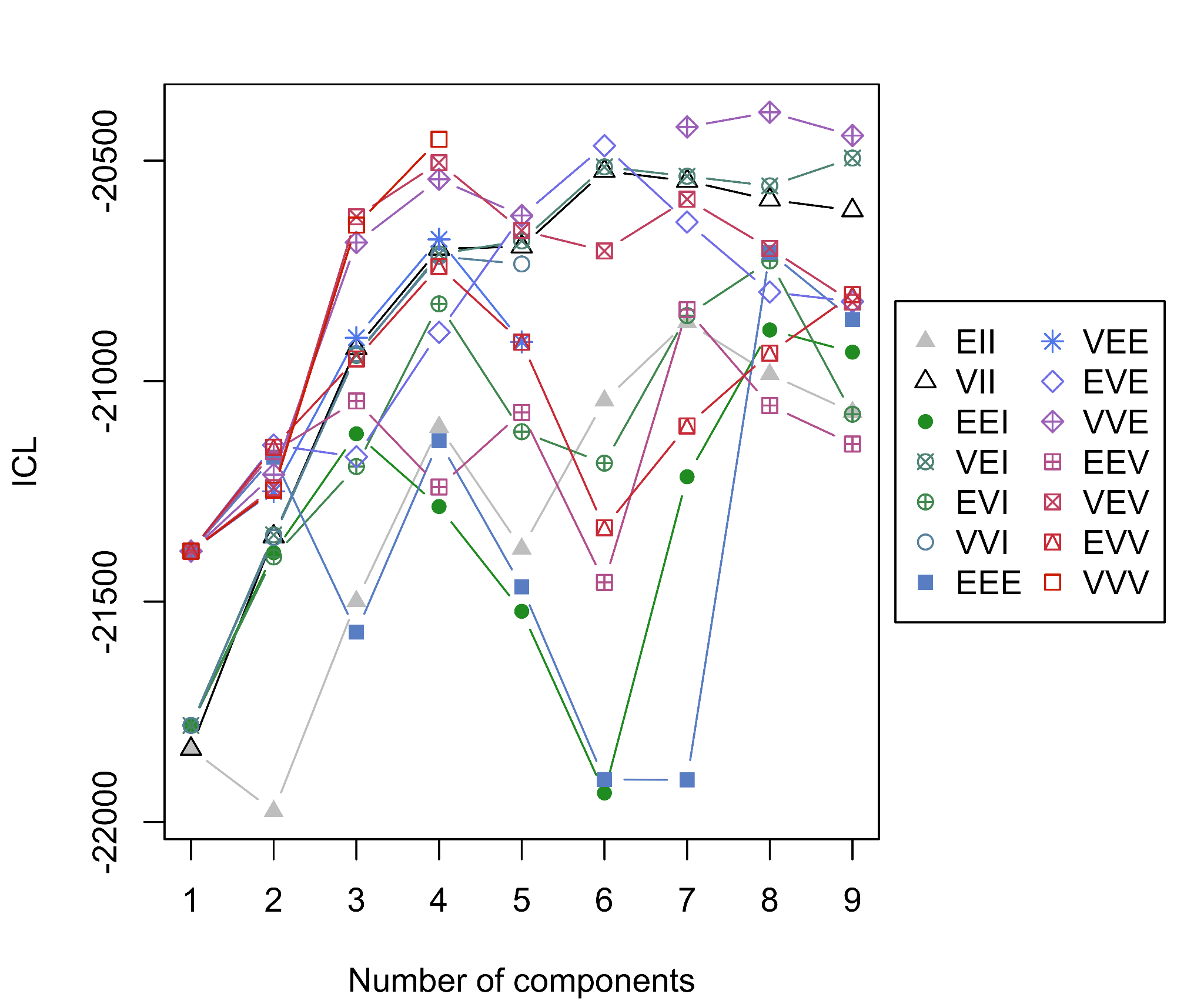
**

**Table S7**

*Cumulative Model Fit Indices for Selected Models and Neighboring Models*

| Number of Profiles | BIC | ICL | BLRT Statistic | BLRT *p* Value | BLRT Meaning | Smallest *n* Proportion |
| --- | --- | --- | --- | --- | --- | --- |
| VEV Model Type | | | | | | |
| 1 | -21385.60 | -21385.60 | 430.51 | < .001 | 2 > 1 | 1.00 |
| 2 | -21013.84 | -21241.59 | 805.87 | < .001 | 3 > 2 | .26 |
| **3** | **-20266.73** | **-20627.22** | **170.96** | < .001 | **4 > 3** | **.31** |
| VVE Model Type | | | | | | |
| 1 | -21385.60 | -21385.60 | 469.53 | < .001 | 2 > 1 | 1.00 |
| 2 | -20967.48 | -21211.98 | 704.77 | < .001 | 3 > 2 | .23 |
| 3 | -20314.12 | -20685.61 | 222.98 | < .001 | 4 > 3 | .31 |
| 4 | -20142.56 | -20542.12 | 87.84 | < .001 | 5 > 4 | .03 |

*Note*. The bolded row indicates the model that we selected. Normally the goal is to minimize BIC values but in the mclust package the BIC value is a negative version of the normal BIC, so in this case the goal is to maximize the BIC value (this applies to ICL values as well, which adds a penalty for entropy to BIC values). The BLRT compares the current model with a model with k+1 profiles, and significant *p* values indicate that the model with k profiles ought to be rejected in favor of the model with k+1 profiles.

**Table S8**

*Cumulative Profiles in Three Different Models*

| Profile |  | Semantic Reliance | | Single Episode Reliance | | Mixed Episodes Reliance | |
| --- | --- | --- | --- | --- | --- | --- | --- |
|  | *n* | *M* | *SD* | *M* | *SD* | *M* | *SD* |
| Two Profile VVE Model | | | | | | | |
| 1 | 359 | 8.62 | 0.99 | 2.04 | 1.16 | 8.56 | 1.01 |
| 2 | 1189 | 6.51 | 2.34 | 5.86 | 2.55 | 6.13 | 2.51 |
| Three Profile VEV Model | | | | | | | |
| 1 | 497 | 8.41 | 1.15 | 2.27 | 1.25 | 8.14 | 1.28 |
| 2 | 572 | 7.29 | 1.27 | 6.99 | 1.10 | 7.51 | 1.26 |
| 3 | 479 | 5.18 | 2.85 | 5.37 | 3.14 | 4.21 | 2.66 |
| Four Profile VVE Model | | | | | | | |
| 1 | 485 | 8.51 | 1.08 | 2.20 | 1.25 | 8.13 | 1.28 |
| 2 | 557 | 7.32 | 1.20 | 6.93 | 1.18 | 7.55 | 1.21 |
| 3 | 458 | 4.74 | 2.52 | 5.98 | 2.76 | 4.68 | 2.64 |
| 4 | 48 | 9.40 | 0.78 | 0.71 | 0.82 | 1.40 | 1.08 |

*Note.* Profile numbers are intended to correspond between models (i.e., the same number represents roughly the same profile across models). Profile 1 roughly corresponds to the low episodic reliance profile in the main text, profile 2 corresponds with the high overall reliance profile, and profile 3 corresponds with the low overall reliance profile.

**Figure S14**

*Cumulative Mean Memory Reliance Ratings in the Two Profile Model*


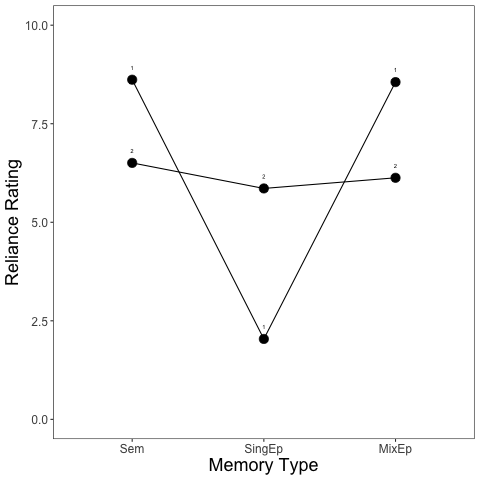


*Note*. Sem = semantic reliance. SingEp = single episode reliance. MixEp = mixed episodes reliance.

**Figure S15**

*Cumulative Mean Memory Reliance Ratings in the (Selected) Three Profile Model*


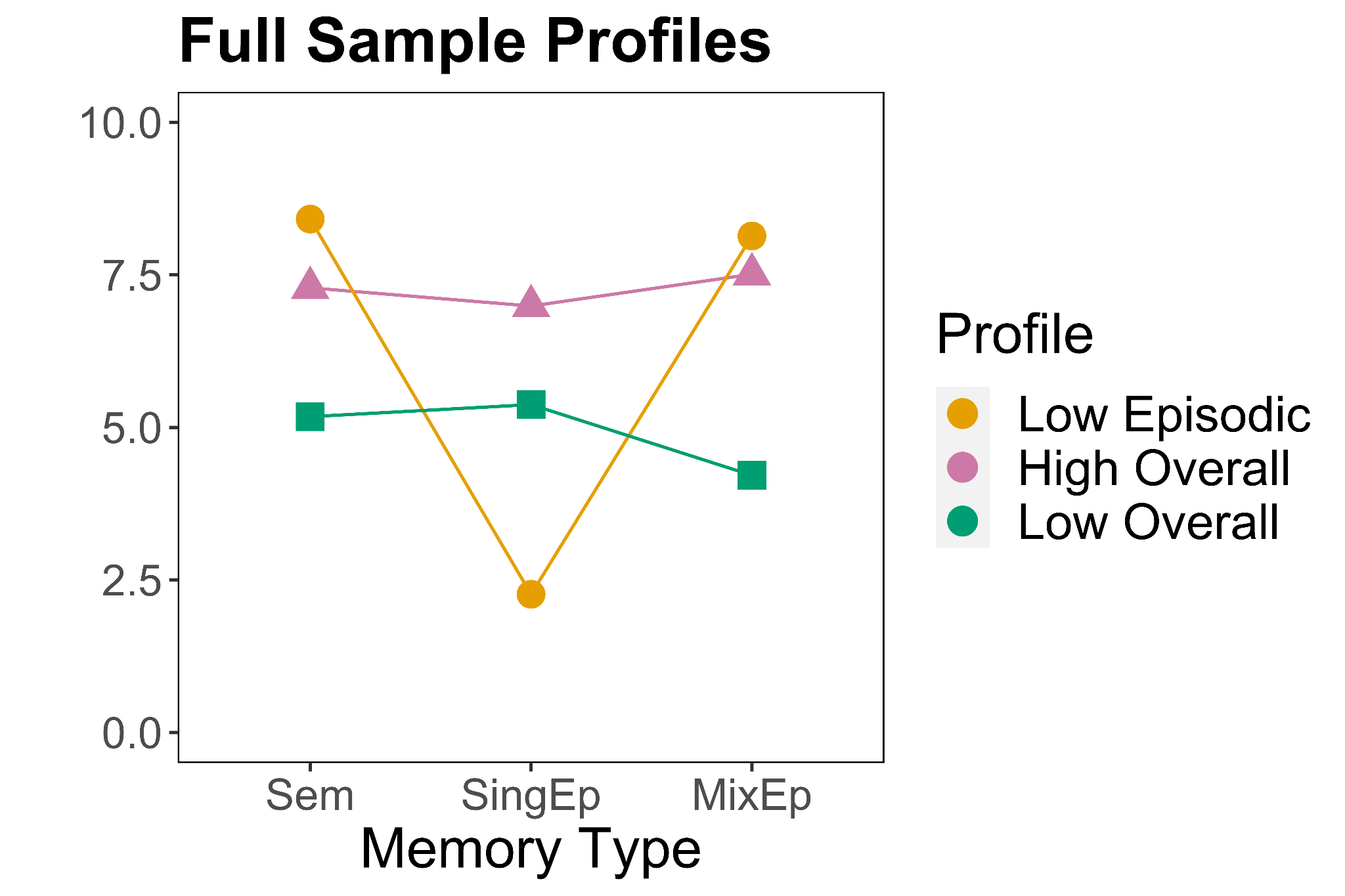


**Figure S16**

*Cumulative Mean Memory Reliance Ratings in the Four Profile Model*

**
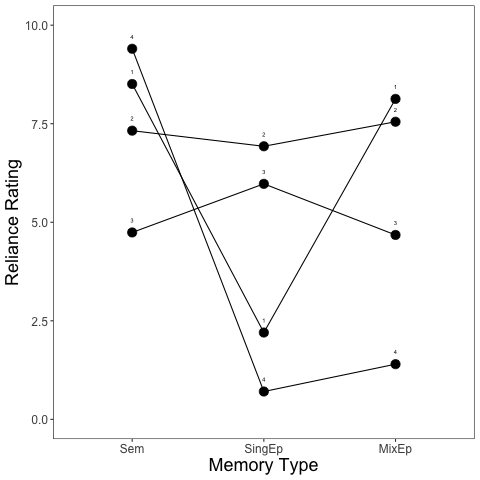
**

*Note*. Sem = semantic reliance. SingEp = single episode reliance. MixEp = mixed episodes reliance.

###

### Cumulative Mixed-Effect Analyses

Table S9 lists cumulative regression statistics for the mixed-effect analyses on the combined (i.e., cumulative) data from Study 1 and Study 2. In line with the main text, we examined whether there are significant differences between the profiles in similarity, similarity of place, similarity of people, vividness, and frequency. We report additional models where we added controls in the analyses of our similarity variables to determine whether one variable in particular might be driving results. The low episodic reliance profile was the main reference group in these analyses, but we also compared the high overall reliance and low overall reliance profiles, using the high overall reliance profile as the reference group. Table S10 depicts the means and standard deviations of these variables across each of the profiles.

**Table S9**

*Study 1 and Study 2 Regression Statistics for Mixed-Effect Models*

| Comparison | *b* | CI | *t* | *p* |
| --- | --- | --- | --- | --- |
| Overall Similarity ~ Profile | | | | |
| Low Epi vs High | -0.21 | [-0.50, 0.08] | -1.45 | .149 |
| Low Epi vs Low | -0.53 | [-0.82, -0.23] | -3.47 | < .001 |
| High vs Low | -0.32 | [-0.60, -0.03] | -2.16 | .031 |
| Overall Similarity ~ Similarity of Place + Profile | | | | |
| SimPl | 0.36 | [0.32, 0.40] | 16.53 | < .001 |
| Low Epi vs High | -0.04 | [-0.31, 0.22] | -0.33 | .745 |
| Low Epi vs Low | -0.19 | [-0.47, 0.09] | -1.32 | .187 |
| High vs Low | -0.14 | [-0.41, 0.12] | -1.06 | .291 |
| Overall Similarity ~ Similarity of People + Profile | | | | |
| SimPpl | 0.10 | [0.06, 0.15] | 4.62 | < .001 |
| Low Epi vs High | -0.16 | [-0.46, 0.15] | -1.01 | .313 |
| Low Epi vs Low | -0.46 | [-0.79, -0.14] | -2.84 | .005 |
| High vs Low | -0.31 | [-0.62, 0.00] | -1.96 | .050 |
| Similarity of Place ~ Profile | | | | |
| Low Epi vs High | -0.53 | [-0.82, -0.23] | -3.48 | < .001 |
| Low Epi vs Low | -0.94 | [-1.25, -0.63] | -5.97 | < .001 |
| High vs Low | -0.42 | [-0.72, -0.12] | -2.73 | .007 |
| Similarity of Place ~ Overall Similarity + Profile | | | | |
| Sim | 0.40 | [0.35, 0.46] | 16.11 | < .001 |
| Low Epi vs High | -0.43 | [-0.71, -0.15] | -3.05 | .002 |
| Low Epi vs Low | -0.75 | [-1.04, -0.46] | -5.04 | < .001 |
| High vs Low | -0.32 | [-0.60, -0.04] | -2.21 | .027 |
| Similarity of Place ~ Similarity of People + Profile | | | | |
| SimPpl | 0.13 | [0.08, 0.17] | 5.28 | < .001 |
| Low Epi vs High | -0.60 | [-0.93, -0.28] | -3.70 | < .001 |
| Low Epi vs Low | -0.89 | [-1.24, -0.55] | -5.15 | < .001 |
| High vs Low | -0.29 | [-0.62, 0.04] | -1.74 | .083 |
| Similarity of People ~ Profile | | | | |
| Low Epi vs High | -0.09 | [-0.47, 0.28] | -0.48 | .633 |
| Low Epi vs Low | -0.62 | [-1.02, -0.22] | -3.05 | .002 |
| High vs Low | -0.53 | [-0.91, -0.14] | -2.70 | .007 |
| Similarity of People ~ Overall Similarity + Profile | | | | |
| Sim | 0.16 | [0.10, 0.23] | 4.77 | < .001 |
| Low Epi vs High | -0.07 | [-0.44, 0.31] | -0.34 | .731 |
| Low Epi vs Low | -0.54 | [-0.94, -0.15] | -2.68 | .007 |
| High vs Low | -0.48 | [-0.85, -0.10] | -2.46 | .014 |
| Similarity of People ~ Similarity of Place + Profile | | | | |
| SimPl | 0.16 | [0.10, 0.22] | 5.19 | < .001 |
| Low Epi vs High | 0.00 | [-0.37, 0.38] | 0.02 | .985 |
| Low Epi vs Low | -0.46 | [-0.86, -0.06] | -2.26 | .024 |
| High vs Low | -0.46 | [-0.84, -0.08] | -2.39 | .017 |
| Arousal ~ Profile | | | | |
| Low Epi vs High | 0.62 | [0.30, 0.95] | 3.84 | < .001 |
| Low Epi vs Low | -0.01 | [-0.34, 0.32] | -0.07 | 0.946 |
| High vs Low | -0.64 | [-0.96, -0.31] | -3.89 | < .001 |
| Frequency ~ Profile | | | | |
| Low Epi vs High | -0.14 | [-0.60, 0.31] | -0.61 | .541 |
| Low Epi vs Low | -0.05 | [-0.52, 0.43] | -0.19 | .847 |
| High vs Low | 0.10 | [-0.36, 0.55] | 0.41 | .681 |
| Vividness ~ Profile | | | | |
| Low Epi vs High | 0.18 | [-0.06, 0.42] | 1.44 | .149 |
| Low Epi vs Low | -0.11 | [-0.36, 0.14] | -0.90 | .371 |
| High vs Low | -0.29 | [-0.53, -0.05] | -2.37 | .018 |

*Note.* We used the following abbreviations in this table: Low Epi = low episodic reliance profile, High = high overall reliance profile, Low = low overall reliance profile, Sim = overall similarity, SimPl = similarity of place, and SimPpl = similarity of people. In the full sample, a total of 239 subjects were eliminated from similarity of people analyses as they indicated they experienced the repeated event alone. A breakdown of sample size by profile for similarity of place analyses was as follows: for the low episodic reliance profile, *n* = 422; for the high overall reliance profile, *n* = 498; for the low overall reliance profile, *n* = 389.

**Table S10**

*Cumulative Means and Standard Deviations of Each Profile Across Five Variables*

| Variable | Low Episodic | | High Overall | | Low Overall | |
| --- | --- | --- | --- | --- | --- | --- |
|  | *M* | *SD* | *M* | *SD* | *M* | *SD* |
| Overall Similarity | 7.21 | 2.13 | 7.03 | 2.13 | 6.74 | 2.68 |
| Similarity of Place | 8.80 | 2.10 | 8.26 | 2.30 | 7.87 | 2.86 |
| Similarity of People | 7.68 | 2.82 | 7.58 | 2.64 | 7.03 | 3.08 |
| Arousal | 5.26 | 2.65 | 5.97 | 2.42 | 5.19 | 2.70 |
| Frequency | 3.09 | 3.80 | 3.05 | 3.67 | 3.39 | 4.12 |
| Vividness | 7.74 | 2.00 | 7.87 | 1.75 | 7.57 | 2.18 |

## Narrative Hypotheses Analysis

In our first preregistration, we predicted that there would be a significant positive correlation between self-reported overall repeated event similarity and the proportion of external content in repeated event narratives (*M* = .39, *SD* = .31). External content usually contains semantic details, other (i.e., metacognitive) details, and/or repetitions, but our preprocessing procedure involved removing other details and repetitions so that external content would correspond with semantic details. We obtained results in the opposite direction to our prediction, and found a significant negative correlation between overall similarity and the proportion of external content in repeated event narratives, *r_rm_*(193) = -.17, [-.29, -.04], *p* = .017. In the context of our study, episodic (i.e., internal) and semantic (i.e., external) content are mutually exclusive and exhaustive, so there was also a corresponding positive correlation between overall similarity and the proportion of episodic content in narratives.

This result was puzzling, and a close examination of the narratives revealed that in some memories, participants would use episodic-like language even when they reported relying very little on a single episode in their recall (e.g., “I click Genshin Impact and the loading screen takes a few seconds and I can hear the loading screen music,” which is an excerpt from a memory with a low single episode reliance score). Thus, many of these recalls appeared to be more of a prototypical reconstruction of the event that were described using episodic-like language, despite not being an actual episode according to the traditional definition (i.e., a single event localized in time and place). Because the automatic scorer was trained on autobiographical interview data (i.e., memories of single events) and not on reports of repeated events specifically, it is possible that in the current form it cannot pick up on linguistic specificities of memory reports of repeated events. Therefore, we decided not to conduct a narrative analysis using the automatic scorer in Study 2.

## Descriptive Results for Content Analysis of Memory Narratives

In accordance with our aim to use our naturalistic samples to inform future repeated events research, we report descriptive results of our content analysis of the memory narratives below. Table S11 depicts the percentages of each topic category in each study, as well as within-topic percentages for each profile. Table S12 depicts the percentage of narratives of social events in the full sample, the topic categories, and the three profiles (see Guidelines for Content Analysis Coding).

**Table S11**

*Percentages of Different Memory Topics in Full Sample and Profiles*

| Study | Full Sample | Low Episodic Reliance (Within-Topic) | High Overall Reliance (Within-Topic) | Low Overall Reliance (Within-Topic) |
| --- | --- | --- | --- | --- |
| Chores | | | | |
| 1 | 13% | 33% | 33% | 33% |
| 2 | 8% | 37% | 37% | 26% |
| Exercise | | | | |
| 1 | 12% | 42% | 39% | 19% |
| 2 | 13% | 28% | 42% | 30% |
| Recreation | | | | |
| 1 | 31% | 32% | 41% | 27% |
| 2 | 37% | 31% | 37% | 32% |
| School | | | | |
| 1 | 23% | 32% | 44% | 24% |
| 2 | 24% | 29% | 41% | 30% |
| Transport | | | | |
| 1 | 7% | 42% | 42% | 16% |
| 2 | 4% | 44% | 20% | 36% |
| Work | | | | |
| 1 | 12% | 44% | 31% | 25% |
| 2 | 10% | 34% | 38% | 28% |
| Miscellaneous | | | | |
| 1 | 2% | 40% | 20% | 40% |
| 2 | 3% | 37% | 29% | 34% |

*Note.* In Study 1, the low episodic reliance profile made up 36% of the sample, the high overall reliance profile was 39% of the sample, and the low overall reliance profile was 25% of the sample. In Study 2, the low episodic reliance profile made up 32% of the sample, the high overall reliance profile was 38% of the sample, and the low overall reliance profile was 31% of the sample. Within-topic profile percentages should be interpreted in relation to these percentages (e.g., a lower-than-expected percentage for low episodic reliance in Study 1 would be less than 36%, while a higher-than-expected percentage would be greater than 36%).

**Table S12**

*Percentages of Social Memories in Full Sample, Within-Topics, and Within-Profiles*

| Variable | Study 1 | Study 2 |
| --- | --- | --- |
| Full Sample | 62% | 65% |
| Chores | 13% | 23% |
| Exercise | 25% | 25% |
| Recreation | 78% | 80% |
| School | 91% | 86% |
| Transport | 32% | 20% |
| Work | 83% | 77% |
| Miscellaneous | 20% | 39% |
| Low Episodic Reliance | 60% | 65% |
| High Overall Reliance | 65% | 69% |
| Low Overall Reliance | 61% | 62% |

# *Note*. Since coding for whether an event was social or individual was binary, the remaining proportions of events were all individual.

# Supplementary References

Fleiss, J. L. (1971). Measuring nominal scale agreement among many raters. *Psychological Bulletin, 76*(5), 378–382. <https://doi.org/10.1037/h0031619>

Gamer, M., Lemon, J., Fellows, I., & Singh, P. (2019). irr: Various coefficients of interrater reliability and agreement. [https://CRAN.R-project.org/package=irr](https://cran.r-project.org/package=irr)

Holm, S. (1979). A simple sequentially rejective multiple test procedure. *Scandinavian Journal of Statistics, 6*(2), 65–70. <http://www.jstor.org/stable/4615733>

R Core Team. (2023). *R: A language and environment for statistical computing*. R Foundation for Statistical Computing, Vienna, Austria. [https://www.R-project.org/](https://www.r-project.org/)

Marks, D. F. (1973), Visual imagery differences in the recall of pictures. *British Journal of Psychology, 64*, 17-24. <https://doi.org/10.1111/j.2044-8295.1973.tb01322.x>

Nylund, K. L., Asparouhov, T., & Muthén, B. O. (2007). Deciding on the number of classes in latent class analysis and growth mixture modeling: A Monte Carlo simulation study. *Structural Equation Modeling, 14*(4), 535–569. <https://doi.org/10.1080/10705510701575396>

Radloff, L. S. (1977). The CES-D Scale: A self-report depression scale for research in the general population. *Applied Psychological Measurement, 1*(3), 385–401. <https://doi.org/10.1177/014662167700100306>

Scrucca, L., Fop, M., Murphy, T. B., & Raftery, A. E. (2016). mclust 5: Clustering, classification and density estimation using gaussian finite mixture models. *The R Journal, 8*(1), 289–317. <https://doi.org/10.32614/RJ-2016-021>

Van Genugten, R. D. I., & Schacter, D. L. (2024). Automated scoring of the autobiographical interview with natural language processing. *Behavior Research Methods, 56*(3), 2243–2259. <https://doi.org/10.3758/s13428-023-02145-x>

Wickham, H., Averick, M., Bryan, J., Chang, W., McGowan, L. D., François, R., Grolemund, G., Hayes, A., Henry, L., Hester, J., Kuhn, M., Pedersen, T. L., Miller, E., Bache, S. M., Müller, K., Ooms, J., Robinson, D., Seidel, D. P., Spinu, V., Takahashi, K., Vaughan, D., Wilke, C., Woo, K., & Yutani, H. (2019). Welcome to the tidyverse. *Journal of Open Source Software, 4*(43), 1686. <https://doi.org/10.21105/joss.01686>

Zsido, A. N., Teleki, S. A., Csokasi, K., Rozsa, S., & Bandi, S. A. (2020). Development of the short version of the Spielberger State—Trait Anxiety Inventory. *Psychiatry Research, 291,* 113223. <https://doi.org/10.1016/j.psychres.2020.113223>
